# Inhibition of histone acetyltransferase function radiosensitizes *CREBBP*/*EP300* mutants via repression of homologous recombination, potentially targeting a gain of function

**DOI:** 10.1038/s41467-021-26570-8

**Published:** 2021-11-03

**Authors:** Manish Kumar, David Molkentine, Jessica Molkentine, Kathleen Bridges, Tongxin Xie, Liangpeng Yang, Andrew Hefner, Meng Gao, Reshub Bahri, Annika Dhawan, Mitchell J. Frederick, Sahil Seth, Mohamed Abdelhakiem, Beth M. Beadle, Faye Johnson, Jing Wang, Li Shen, Timothy Heffernan, Aakash Sheth, Robert L. Ferris, Jeffrey N. Myers, Curtis R. Pickering, Heath D. Skinner

**Affiliations:** 1 Department of Biochemistry, All India Institute of Medical Sciences (AIIMS), Bilaspur, Himachal Pradesh India; 2 Department of Radiation Oncology, University of Pittsburgh, UPMC Hillman Cancer Center, Pittsburgh, PA USA; 3 Department of Experimental Radiation Oncology, University of Texas, MD Anderson Cancer Center, Houston, TX USA; 4 Department of Head and Neck Surgery, University of Texas, MD Anderson Cancer Center, Houston, TX USA; 5 Department of Otolaryngology-Head & Neck Surgery, Baylor College of Medicine, Houston, TX USA; 6 TRACTION Platform, University of Texas, MD Anderson Cancer Center, Houston, TX USA; 7 Department of Radiation Oncology, Stanford University, Stanford, CA USA; 8 Department of Thoracic and Head and Neck Medical Oncology, University of Texas, MD Anderson Cancer Center, Houston, TX USA; 9 The University of Texas Graduate School of Biomedical Sciences, Houston, TX USA; 10 Department of Biostatistics, University of Texas, MD Anderson Cancer Center, Houston, TX USA; 11 Department of Medicine, Baylor College of Medicine, Houston, TX USA; 12 Department of Otolaryngology, University of Pittsburgh, UPMC Hillman Cancer Center, Pittsburgh, PA USA

**Keywords:** Cancer genomics, Cancer therapy, Tumour biomarkers

## Abstract

Despite radiation forming the curative backbone of over 50% of malignancies, there are no genomically-driven radiosensitizers for clinical use. Herein we perform in vivo shRNA screening to identify targets generally associated with radiation response as well as those exhibiting a genomic dependency. This identifies the histone acetyltransferases *CREBBP*/*EP300* as a target for radiosensitization in combination with radiation in cognate mutant tumors. Further in vitro and in vivo studies confirm this phenomenon to be due to repression of homologous recombination following DNA damage and reproducible using chemical inhibition of histone acetyltransferase (HAT), but not bromodomain function. Selected mutations in *CREBBP* lead to a hyperacetylated state that increases CBP and BRCA1 acetylation, representing a gain of function targeted by HAT inhibition. Additionally, mutations in *CREBBP*/*EP300* are associated with recurrence following radiation in squamous cell carcinoma cohorts. These findings provide both a mechanism of resistance and the potential for genomically-driven treatment.

## Abstract (web summary)

Mutations in histone acetyltransferases (HATs) CREBBP and EP300 are generally thought to lead to decreased function or absence of protein product. Here the authors describe a gain of function of several CREBBP mutations leading to baseline hyper-acetylation, increased homologous recombination and potential synergy between radiation and HAT inhibition in CREBBP/EP300 mutant tumors.

## Introduction

With a few isolated exceptions, the cure of solid tumors requires effective local therapy. In the vast majority of disease sites, this translates to a need for radiation, either alone or as part of a treatment package, including surgery. Despite the large number of targeted and immunotherapies introduced over the past decade or more, in nearly all cases, the only agents available to improve responses to radiation are the cytotoxic chemotherapies that have been in use since the 1980s or earlier. Because of this, an effective and minimally toxic radiosensitizer has the potential to, in short order, positively impact hundreds of thousands of patients.

One exemplar of this phenomenon is head and neck squamous-cell carcinoma (HNSCC), the curative treatment of which has remained largely unchanged over the past two decades. While ~75% of patients with HNSCC require radiation for the treatment of their disease, the recent failure of cetuximab means there are generally no biologically driven radiosensitizers available to improve response and decrease toxicity of this therapy^1–4^. Nor has the advent of immunotherapy changed the current paradigm, with a recent clinical trial of chemoradiation combined with immunotherapy closed due to lack of efficacy^5^.

Despite these failures, the search for improved combinations with radiation remains critical. Again, focusing solely on the close to 300,000 patients with HNSCC annually worldwide who are recommended to receive radiation, an agent that improves the efficacy of this treatment by 15% could lead to more than as 30,000 lives saved annually^6,7^.

However, most antineoplastic agents tested in the preclinical setting ultimately fail to be translated to the clinic due to multiple factors, including the artificial nature of in vitro systems and unforeseen toxicity^8,9^. Targets identified as radiosensitizers in an in vitro model, may underperform in vivo due to complex interactions within the tumor itself. Additionally, the same microenvironment interactions could be potential targets for radiosensitization and may not be readily identified using in vitro screening techniques.

Additionally, most large-scale screening approaches using cell lines with known genomic status have not exposed the cells to radiation and, thus, have not identified tumor mutations or alterations that may be associated with specific targets for radiosensitization^10^. The model of a genomically mediated “Achilles’ heel” in tumors harboring a specific genotype has perhaps best been characterized for BRCA1-altered tumors and their dramatic response to PARP inhibition^11^. However, this model can be expanded to the combination of novel agents with DNA-damaging therapies in specific genetic backgrounds^12^. This is highly advantageous as genomically driven radiosensitizers have the potential to only affect the mutated cancer cells, and not normal cells, providing improved responses with less toxicity.

In the current investigation, we begin with an in vivo screening analysis to identify radiosensitizers in HNSCC, as well as a further analysis to identify potential targets based on somatic mutation. These data lead to the identification of histone acetylation as a target, potentially driven by a gain of function in certain classes of HAT/TAZ2-domain mutants. These mutations exhibit reduced basal-inhibitory function, leading to a hyperacetylated state and potential dependency on homologous recombination and BRCA1 for DNA-damage repair. The importance of *CREBBP* and *EP300* mutation is underscored following analysis of tumor tissues in several cohorts of patients with SCC of the head and neck, lung, or cervix treated with radiation therapy, identifying these mutations as associated with radioresistance and poor outcome.

## Results

### In vivo screening identifies multiple potential radiosensitization targets

We performed in vivo shRNA library screens in tumors generated from 5 HNSCC cell lines of varying genetic background and HPV status (HPV-positive: UM-SCC-47 and UPCI:SCC-152), (HPV-negative: UM-SCC-22a, HN31, and Cal 27) treated with radiation (Supplementary Table 1). Two libraries were used: one encompassing most genes known to be targeted by available antineoplastic agents currently in clinical use or in clinical trials and the second targeting the DNA-damage repair pathway (Supplementary Table 2).

**Fig. 1:**
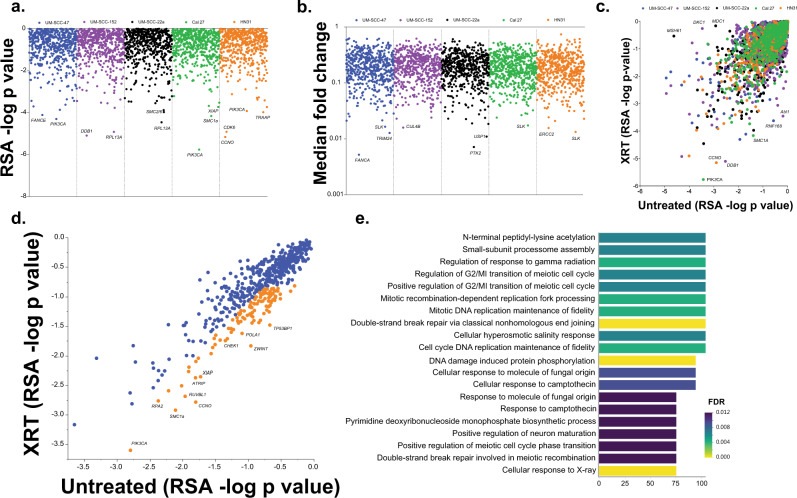
In vivo shRNA screening for radiosensitizing targets. **a** & **b** RSA -log *p*-values (**a**) and median fold change (**b**) for each target in our two shRNA screening libraries for each tumor type tested. **c** & **d** RSA -log *p*-values in irradiated (XRT, y axis) versus untreated (x axis) for each tumor type tested (**c**) and the average for each group (**d**). Target genes in orange were selected for further analysis as potential radiosensitizers and analyzed for Gene Ontology term enrichment (**e**) (two-sided *p*-value presented).

To determine a gene-level summary estimate of the impact of knockout of each gene, we performed redundant shRNA analysis (RSA) to generate log *p*-values for each gene for irradiated tumors (Fig. 1a). Additionally, quantile-transformed median fold change (fc) for each target was analyzed (Fig. 1b). This analysis identified several targets known to be associated with radiosensitization, such as *CDK6*, *XIAP*, *PIK3CA*, and *PTK2*^13–17^. However, to further identify targets specific for radiation response, as opposed to those primarily inhibiting tumorigenesis, we evaluated RSA -log p values for irradiated compared with untreated tumors in complimentary experiments reported elsewhere^18^ (Fig. 1c, d). We examined the average for each group and selected targets highlighted in orange in Fig. 1d, based on values favoring radiation response versus effects on tumorigenesis, defined very broadly to maximize potential target identification (Supplementary Table 3 for selected targets). In this group, we identified multiple DNA-damage repair genes, such as *TP53BP1, ATRIP*, and *RUVBL1*. Additionally, inhibition of *XIAP* and *PIK3CA* was highly associated with radioresponse, even above their antitumor effects.

Gene ontology (GO) analysis of targets identified in Fig. 1d was then performed (Fig. 1e) and, as expected based on the targets screened, DNA-damage repair processes were highly represented in this analysis. However, several additional pathways, particularly protein lysine acetylation, were also identified.

### *CREBBP*, *EP300*, or dual-specificity protein-kinase (TTK) inhibition in combination with radiation in *CREBBP*/*EP300-*mutated tumors leads to radiosensitization

**Fig. 2:**
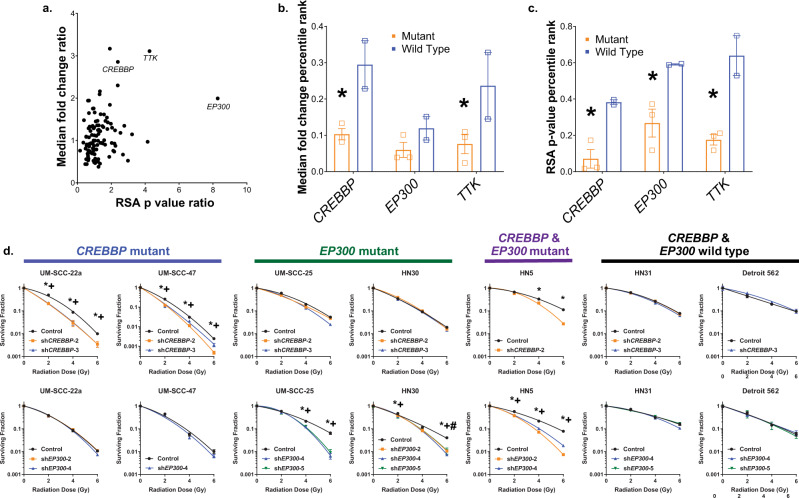
In vivo screening identifies genomically associated radiosensitization in *CREBBP*/*EP300*-mutated tumors. **a** Ratio of *CREBBP* mutant vs. wild type for target fold change (y axis) and RSA log *p*-value (x axis) for radiosensitizing targets selected from Fig. 1. **b**, **c** Difference between *CREBBP*/*EP300* mutant (*n* = 3) and wild-type (*n* = 2) tumors from the in vivo shRNA study as a function of target fold change (**c**) and RSA log *p*-value (**c**). (*)—two-sided *p* < 0.05 versus wild type. **d** Clonogenic assays following irradiation of HNSCC cell lines expressing control and either *CREBBP* or *EP300* shRNA using a minimum of 3 independent samples for each condition and are presented as mean values +/−SEM. In B–D, comparisons were evaluated using ANOVA with post hoc analysis adjusted for multiple comparisons. For (*) sh*CREBBP*-2 and (#) sh*CREBBP*-3, *p* < 0.05 versus control. All *p*-values two-sided.

In addition to finding general radiosensitization targets, we wished to determine if specific somatic mutations observed in HNSCC were associated with targets, with a goal of identifying genomically associated radiosensitizers. To accomplish this, we compared the existing RSA log *p*-values and median fold change data for radiosensitizing targets between tumors that are wild type or mutant for somatic mutations that are represented by the models in the study. This was defined as at least 2 of 5 models harboring a mutation that is observed in >10% of HNSCC. The specific comparisons were: (i) *CREBBP*/*EP300* (HN31 and CAL 27 vs. UM-SCC-47, UPCI:SCC-152 and UM-SCC-22a), (ii) *NOTCH1* (HN31, UM-SCC-47, and UM-SCC-22a vs. CAL 27 and UPCI:SCC-152), and (iii) *CASP8* (UM-SCC-47, UM-SCC-22a and CAL 27 vs. HN31 and UPCI:SCC-152) (Fig. 2a–c).

While no differences were observed in the comparison of *CASP8* wild-type and mutant tumors in either of the libraries, several targets seemed to preferentially increase sensitivity to radiation in *NOTCH1* and *CREBBP* mutant tumors (Fig. 2a and Supplementary Fig. 1). Inhibition of *Abl1*, *CCNO*, and *KDM1a* appeared to preferentially radiosensitize *NOTCH1* mutant tumors (Supplementary Fig. 1), while inhibition of the *CREBBP* and *EP300* genes, as well as the dual-specificity protein kinase (*TTK*), was associated with increased in vivo sensitivity to radiation in the *CREBBP* mutant tumors in this screen (Fig. 2a–c).

### Inhibition of *CREBBP* or *EP300* expression leads to in vitro radiosensitization, but only in the presence of cognate mutations

Based on the degree of effect as well as the identification of lysine acetylation as an enriched pathway in radiosensitizing targets, we chose *CREBBP* and *EP300* for further validation by performing targeted knockdown (KD) across an expanded set of cell lines. We utilized shRNA KD to either *CREBBP* or *EP300* in HNSCC cell lines (Fig. 2d and Supplementary Fig. 2) of varying *CREBBP*/*EP300* mutation status (see Supplementary Table 1 for details). These cell lines were then treated with radiation, and clonogenic survival was assayed (Fig. 2d). Similar to our in vivo screening results, *CREBBP* or *EP300* KD was associated with increased sensitivity to radiation. However, the sensitivity appeared only in the context of a mutation in the cognate gene. For example, *CREBBP* KD, but not *EP300*, led to significant radiosensitization in the *CREBBP* mutant cell line UM-SCC-22a, which is *EP300* wild type. Similarly, *EP300* KD, but not *CREBBP*, led to significant radiosensitization in the *EP300* mutant cell line UM-SCC-25 that is wild type for *CREBBP*. This pattern was consistently observed over all cell lines tested (Fig. 2d).

### *CREBBP* inhibition leads to increased apoptosis and decreased BRCA1 foci formation following radiation in mutant cells

**Fig. 3:**
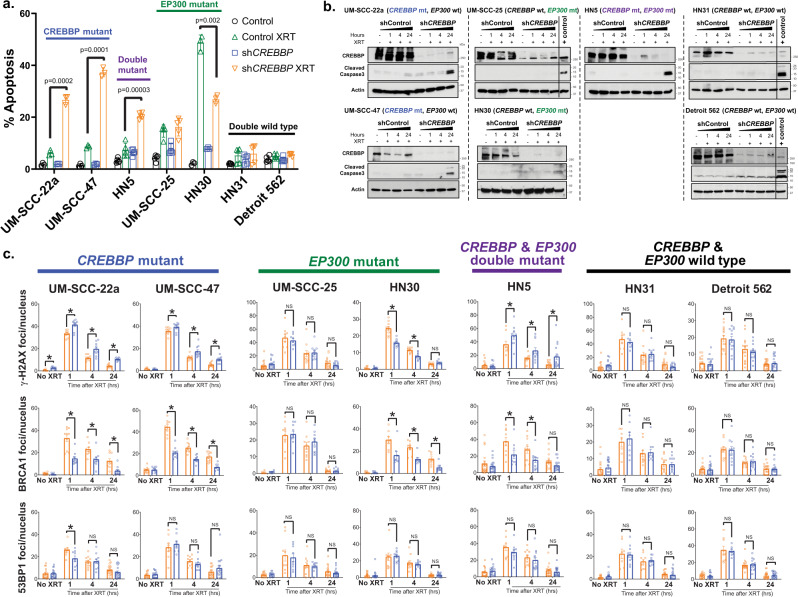
Impaired HR and increased apoptosis is observed following CBP inhibition in *CREBBP* mutant cells. **a**, **b** TUNEL staining (**a**) and caspase-3 immunoblot (**b**) following irradiation in HNSCC cells expressing control or *CREBBP* shRNA. **c** ɣ-H2AX, 53BP1, and BRCA1 foci following irradiation in shControl or sh*CREBBP* HNSCC cells. Significance tested via ANOVA with post hoc analysis adjusted for multiple comparisons. For **a**, **c**, a minimum of three independent samples for each condition are shown and are presented as mean values +/− SEM. For A, two-sided *p*-value shown for each indicated comparison. For **c**, (*) indicates a two-sided *p* < 0.05 for the comparisons shown.

To further evaluate the observed radiosensitization, we examined apoptosis in multiple cell lines expressing shRNA to *CREBBP* (Fig. 3a, b). The combination of *CREBBP* inhibition and radiation led to dramatically increased TUNEL staining in *CREBBP* mutant (but not wild type) cell lines (Fig. 3a). Similar results were observed on immunoblot examining caspase-3 cleavage (Fig. 3b). We also examined the DNA-damage response via immunofluorescence staining of DNA-damage foci (Fig. 3c) (representative images in Supplementary Fig. 3a). Importantly, ɣ-H2AX foci, a marker of DNA damage, were increased following *CREBBP* KD and radiation in all 3 *CREBBP* mutant cell lines examined. In contrast, under the same conditions, BRCA1 foci induction was significantly reduced in all 3 *CREBBP* mutant cell lines and HN30 (Fig. 3c). In the HN30 line ɣ-H2AX induction was reduced following radiation and *CREBBP* KD, likely indicating a different biology occurring in this *TP53* and *CREBBP* WT cell line. Irrespective of mutational status, inhibition of *CREBBP* generally had little effect on 53BP1 foci formation following radiation (Fig. 3c). *CREBBP* inhibition also had minimal effects on BRCA1 transcription or cell cycle distribution at baseline or following radiation, regardless of mutation status (Supplementary Fig. 3b–d).

### Knockdown of *CREBBP* leads to dramatic in vivo radiosensitization

**Fig. 4:**
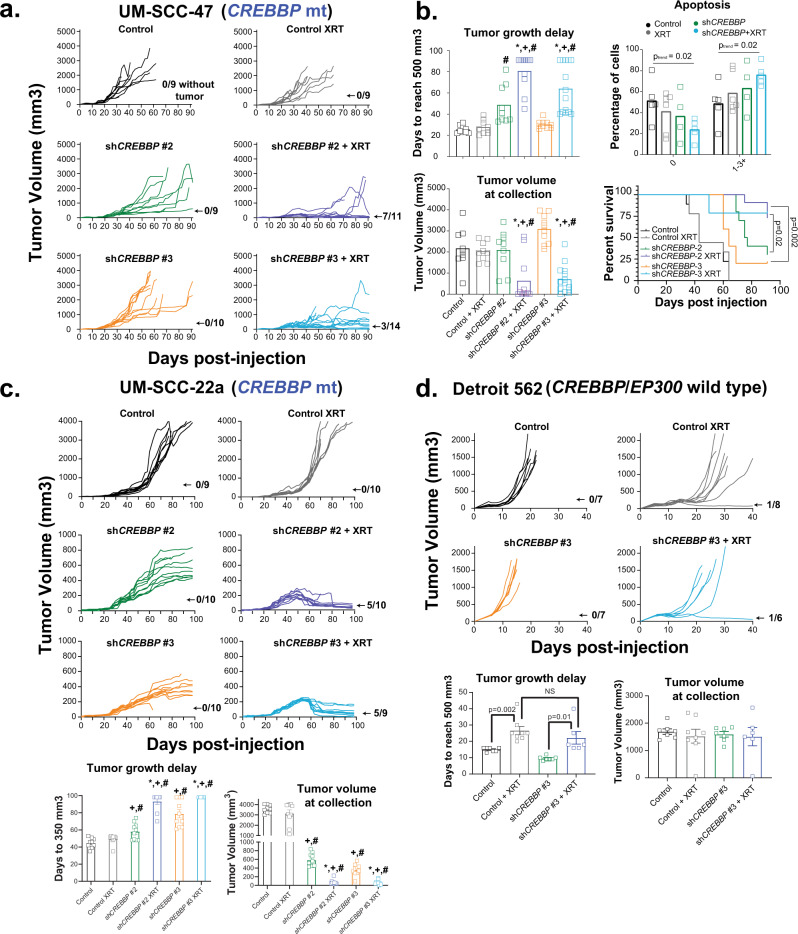
Inhibition of CBP in *CREBBP* mutant tumors leads to radiosensitization in mutant but not wild-type in vivo HNSCC models. **a** Individual tumor-growth curves in a UM-SCC-47 xenograft model expressing either control or two different *CREBBP* shRNAs following irradiation at 2 Gy/day for eight days. **b** Tumor-growth delay (to 500 mm3), tumor volume at collection, survival (all with n per group shown in panel **a**), and in vivo apoptosis (via TUNEL assay) (*n* = minimum of 4) in UM-SCC-47 xenograft tumors. Tumors for TUNEL assay were collected 8 h following the final dose of radiation in a concurrent experiment with tumor growth delay and survival. Log-rank statistics comparing sh*CREBBP*-2 XRT or sh*CREBBP*-3 XRT groups versus unirradiated control for each group were performed (two-sided *p*-value shown). **c** Individual growth curves, tumor-growth delay, and tumor volume at collection from a similar experiment performed in the UM-SCC-22a xenograft model. Of note, tumor-growth delay (*n* as shown in the tumor growth panel) to 350 mm^3^ was used for this experiment as many tumors in the treatment groups did not reach 500 mm^3^. **d** Individual growth curves, tumor-growth delay, and tumor volume at collection (n for both shown in the growth-curve panel) from a similar experiment in the Detroit 562 xenograft model. For bar graphs, data are presented as mean values +/− SEM. Significance tested via ANOVA with post hoc analysis adjusted for multiple comparisons. Two-sided *p* < 0.05 as indicated or compared with unirradiated sh*CREBBP* (*), unirradiated control (+), irradiated control (#).

We further evaluated the therapeutic potential of targeting *CREBBP* using three separate in vivo models of HNSCC, two harboring mutant *CREBBP* and one with wild-type *CREBBP*. In the first study, we used the *CREBBP* mutant cell line UM-SCC-47 to generate tumors in the mouse flank. Tumors were treated with 2 Gy x 8 days, in a fractionation scheme designed to recapitulate that used in patients, albeit to a much lower dose (16 Gy total vs. 70 Gy in the clinic). In this experiment, radiation or *CREBBP* KD (using two distinct shRNA constructs) alone had minimal-to-modest effect; however, the combination led to a profound tumor-growth delay, decreased tumor volume, and improved survival (Fig. 4a, b). Indeed, at the conclusion of the tumor growth delay experiment, seven tumors in the irradiated sh*CREBBP*-2 group (64%) and three tumors in the irradiated sh*CREBBP*-3 group (21.4%) had regressed below the limits of detection. An additional separate experiment was performed to measure apoptosis. TUNEL staining increased in the combined sh*CREBBP* and radiation groups 8 h following the final dose of radiation (2 Gy x 8 d) compared with both the irradiated shControl tumors and unirradiated *CREBBP* knockdown tumors (Fig. 4b). We performed a similar experiment using tumors derived from cells of another *CREBBP* mutant line, UM-SCC-22a (Fig. 4c). While inhibition of *CREBBP* alone had a significant effect in this model, we again observed a profound radiosensitization compared with the unirradiated control tumors. In this model, virtually all tumors dramatically regressed, and most tumors regressed below the limits of detection.

We additionally performed a similar experiment using tumors derived from the *CREBBP* wild-type cell line Detroit 562 (Fig. 4d). In this model, treatment with 2 Gy x 8 days led to significant tumor-growth delay in the control and sh*CREBBP* tumors. However, inhibition of *CREBBP* led to no significant radiosensitization. Specifically, tumor-growth delay was slightly (although not significantly) worse with *CREBBP* knockdown, with no effect on final tumor volume.

### Inhibition of CBP and p300 histone acetyltransferase (HAT), but not bromodomain function, leads to radiosensitization in *CREBBP*/*EP300* mutants associated with repression of HR

To further examine the therapeutic relevance of the observed radiosensitization in HNSCC, we utilized several chemical inhibitors of CBP and/or p300 function: (1) ICG-001, a CBP-specific inhibitor that is thought to inhibit the interaction between CBP and β-catenin, although it is also known to have β-catenin-independent effects (note: PRI-724 is an active enantiomer of ICG-001)^19–21^; (2) GNE-272, a bromodomain-specific inhibitor for both CBP and p300^22^; (3) A-485, a histone acetyltransferase inhibitor specific for CBP and p300 (note: A-486 is an inactive analog and used as a negative control)^23^. Similar to shRNA-based inhibition of *CREBBP*, ICG-001 led to significant in vitro radiosensitization on clonogenic assay, but only in those cell lines harboring *a CREBBP* mutation (Supplementary Fig. 4a, b). Although the analog of ICG-001, PRI-724, is actively in clinical trial development, neither of these agents target p300, and as predicted, we generally did not observe similar sensitization in a wild type or an *EP300* mutant cell line (Supplementary Fig. 4a, b).

**Fig. 5:**
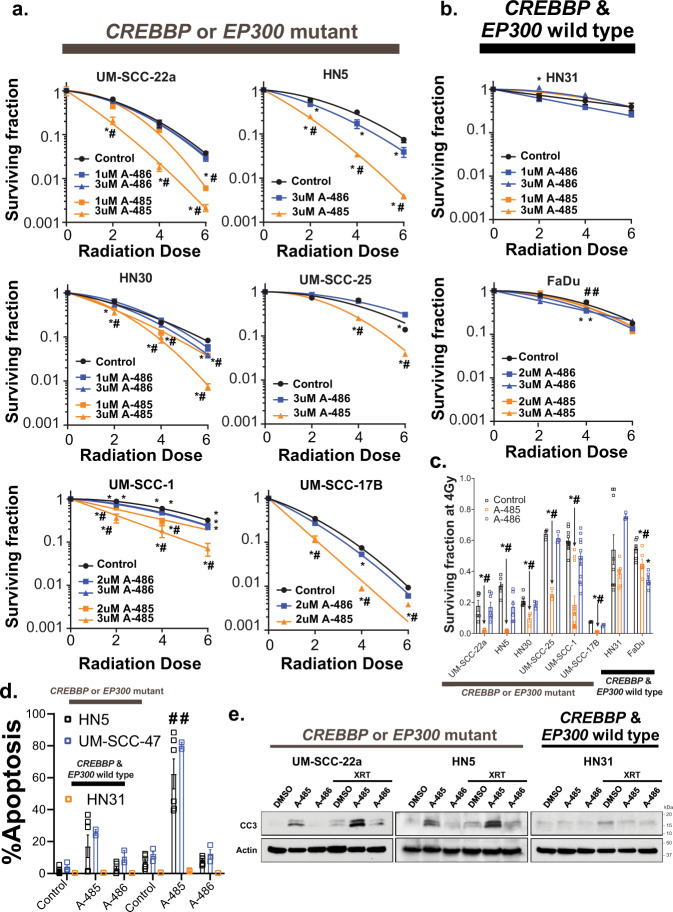
The HAT inhibitor A-485 radiosensitizes cells harboring *CREBBP* and *EP300* mutations. **a**, **b** Clonogenic survival following irradiation and either A-485 (active) or A-486 (inactive) in *CREBBP*/*EP300* mutant (**a**) or wild-type (**b**) HNSCC cells. **c** Surviving fraction at 4 Gy for each cell line (highest dose of A-485/A-486 shown). **d**, **e** TUNEL assay (**d**) and caspase 3 cleavage (**e**) in UM-SCC-47 (*CREBBP* mut), and HN5 (*CREBBP*/*EP300* mut) and HN31 (*CREBBP*/*EP300* wt) cells treated with radiation and either A-485 (active) or A-486 (inactive). For **a**–**e**, a minimum of three independent samples for each condition are shown and are presented as mean values +/− SEM with (*, #) -*p* < 0.05 vs. control (*) and A-486 (#). *p*-values are two-sided and derived from ANOVA with post hoc analysis adjusted for multiple comparisons.

Thus, to maximize the clinical impact of the observed radiosensitization, we examined additional inhibitors, currently in clinical development, that inhibit both CBP and p300 function. We initially tested GNE-272, a bromodomain inhibitor, however, this agent had minimal effects on sensitivity to radiation on clonogenic assay (Supplementary Fig. 5), independent of *CREBBP* or *EP300* status. However, the HAT inhibitor A-485, but not the inactive A-486 analog, led to a profound radiosensitization in cell lines harboring a mutation in either *CREBBP* or *EP300* (Fig. 5a, c), but not in wild-type cell lines (Fig. 5b, c). The observed radiosensitization was largely due to increased apoptosis following the combination of A-485 and radiation (Fig. 5d, e).

**Fig. 6:**
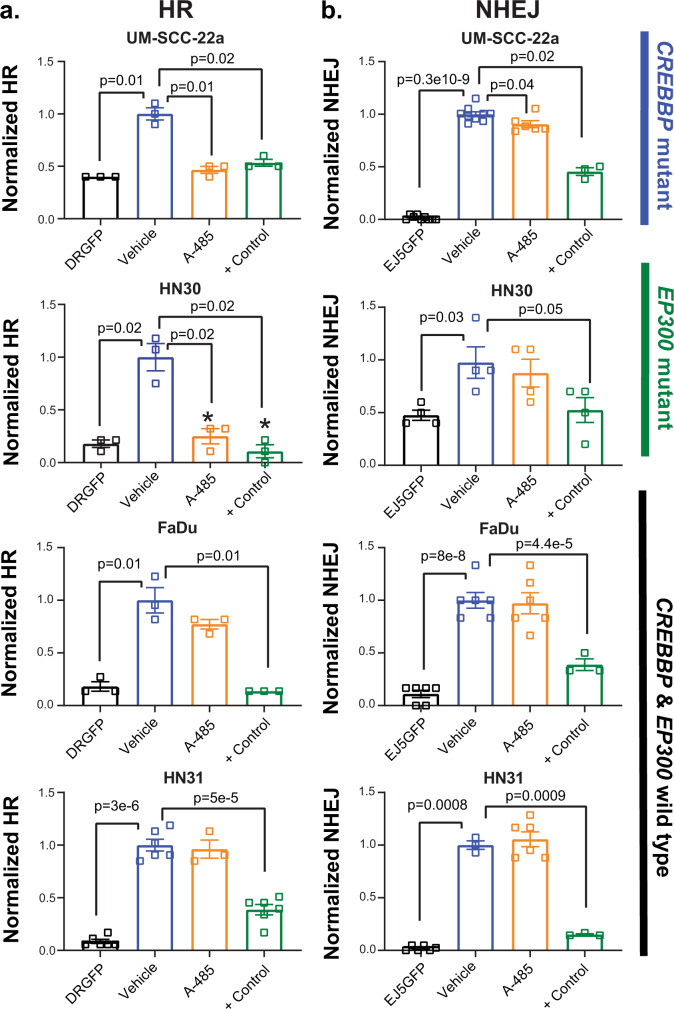
HAT inhibition primarily reduces HR and not NHEJ in *CREBBP*/*EP300* mutant cells. **a**, **b** I-Scel assay for HR (**a**) and NHEJ (**b**) as described in the methods following treatment with A-485, A-486, or + control (ATMi or ATRi). A minimum of three independent samples for each condition are shown and are presented as mean values +/− SEM. *p*-values for each indicated comparison are two-sided and derived from ANOVA with post hoc analysis adjusted for multiple comparisons.

Because of the observed effects of sh*CREBBP* on BRCA1 foci formation following radiation, and the relationship between BRCA1 and homologous recombination (HR), we wished to evaluate the relationship between HAT inhibition and homologous recombination directly via I-Scel-based assay^24^. We utilized both HNSCC and lung-cancer cell lines (which also harbor mutations in either *CREBBP* or *EP300*) (Supplementary Table 1). Similar to HNSCC lines, treatment with A-485 generally led to more profound radiosensitization in *CREBBP*/*EP300* mutant but not wild-type lung-cancer cell lines (Supplementary Fig. 6a, b). Additionally, A-485 led to dramatic and significant repression (ranging from 44% to 75% versus control) of HR in *CREBBP* or *EP300* mutant HNSCC and lung-cancer cell lines, but not in wild-type cells (Fig. 6a) (Supplementary Fig. 6c, d). Conversely, A-485 had little effect on NHEJ in HNSCC cell lines (Fig. 6b).

### The observed radiosensitization in *CREBBP*/*EP300* mutants is not *CREBBP* or *EP300* expression-level dependent

One potential explanation for the observed radiosensitization is a simple dose-dependency. Namely, if basal levels of *CREBBP* or *EP300* are significantly diminished in mutant cells (and tumors), a more profound inhibition is possible, leading to a more pronounced phenotype. Thus, the observed effect could be due to a more complete inhibition of the protein in the setting of a loss-of-function mutation. To further explore this hypothesis, we examined basal expression in HNSCC cell lines and tumors in the context of various *CREBBP* and *EP300* mutations. Interestingly, basal *CREBBP* and *EP300* gene expression in the cell lines used in this study was not directly associated with the underlying mutation (Supplementary Fig. 7a). Moreover, in a panel of 82 HNSCC cell lines, neither *CREBBP* nor *EP300* mutation was directly associated with mRNA expression^25^ (Supplementary Fig. 7b).

In clinical samples from the TCGA HNSCC cohort, no significant difference in *CREBBP* (Supplementary Fig. 7c) or *EP300* (Supplementary Fig. 7d) gene expression was observed in mutant tumors. Moreover, even in the context of nearly complete inhibition of CBP protein expression (Fig. 3b), neither HN31 nor HN30 (*CREBBP* wild-type cell lines) were sensitized to radiation (Fig. 2d). Conversely, incomplete inhibition of *EP300* (in the case of *EP300* mutants HN5 and HN30) led to significant radiosensitization (Fig. 2d and Supplementary Fig. 2). Collectively based on these data, the presence of radiosensitization in mutant tumors (and its absence in wild-type tumors) does not appear to be directly related to total amount of either *CREBBP* or *EP300* present.

### Histone acetylation is associated with radiosensitization in *CREBBP*/*EP300* mutants following CBP/p300 targeting in HNSCC

**Fig. 7:**
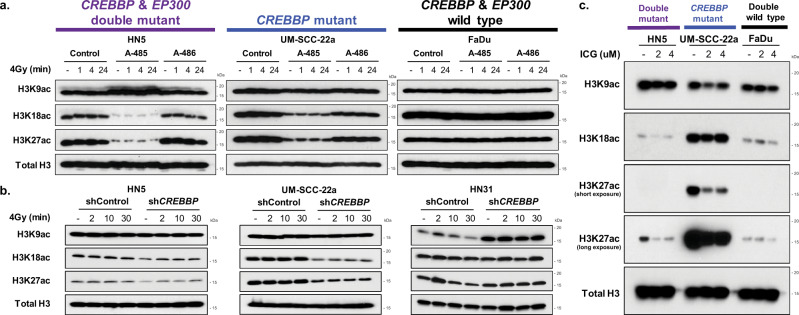
Cells with mutations in *CREBBP* exhibit inhibition of histone acetylation following inhibition of CBP function. **a**–**c** Histone acid extraction and immunoblot following A-485 (**a**), sh*CREBBP* (**b**), or ICG-001 (**c**) in HNSCC cell lines of varying *CREBBP* and *EP300* status.

Based on the observed sensitization following HAT, but not bromodomain inhibition, and the lack of evidence for this phenomenon being solely gene-expression dependent, we further investigated histone-acetylation status in HNSCC cell lines following combination treatment. As expected, treatment with A-485 inhibited histone acetylation at H3K18 and H3K27 in HN5 and UM-SCC-22a cells (Fig. 7a). Both cell lines harbor mutations in either *CREBBP* and/or *EP300* and exhibit sensitivity when HAT inhibition is combined with radiation. Interestingly, FaDu, *a CREBBP*/*EP300* wild-type cell line with no observed radiosensitization, showed no effect on histone acetylation following treatment with HAT inhibitor at baseline or following radiation treatment. Similar effects on histone acetylation were observed following the expression of shRNA specific to *CREBBP* (Fig. 7b) and treatment with ICG-001, although a slight decrease in acetylation was observed in FaDu cells following this latter treatment (Fig. 7c).

### Selected mutations in *CREBBP* exhibit increased acetylation activity consistent with gain of function and potentially mediating response to radiation

**Fig. 8:**
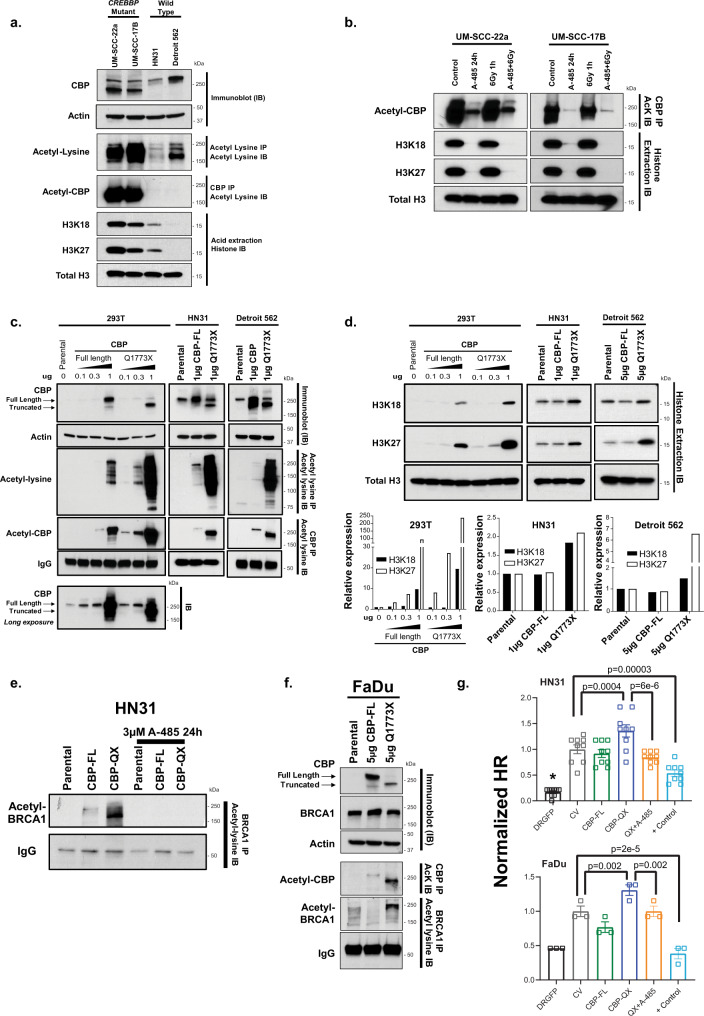
Gain-of-function mutation in *CREBBP* mutant cells. **a**–**f** Immunoblot (IB), histone acid extraction or immunoprecipitation (IP) for CBP, acetyl lysine, or BRCA1 was performed as indicated in the individual panels. **a** Global protein acetylation (IP acetyl lysine and IB for acetyl lysine), CBP autoacetylation (IP CBP and IB for acetyl lysine), and histone acetylation (acid extraction and IB) in HNSCC cell lines expressing wild-type *CREBBP* (HN31, Detroit 562) or *CREBBP* harboring a mutation in the TAZ2 domain (UM-SCC-22a, UM-SCC-17b). **b** CBP auto- and histone acetylation following treatment with A-485 in *CREBBP* mutant lines. **c**, **d** Global acetyl lysine and CBP autoacetylation (**c**), as well as histone acetylation (**d**) (densitometry below blot) in 293 T, HN31, and Detroit 562 cells forced to express either full-length CBP or a representative TAZ2 mutant CBP (Q1773X). **e** BRCA1 acetylation in HN31 cells forced to express full-length or TAZ2 mutant CBP and treated with A-485. **f** Total and acetyl CBP and BRCA1 in FaDu cells forced to express full-length or TAZ2 mutant CBP. **g** HR assay in either HN31 or FaDu cells forced to express full-length or TAZ2 mutant CBP. A minimum of three independent samples for each condition are shown and are presented as mean values +/− SEM. Two-sided *p*-values for each indicated comparison are derived from ANOVA with post hoc analysis adjusted for multiple comparisons.

Previously, mutations within the inhibitory TAZ domain of CBP have been linked to increased histone acetylation^26^. Because of this observation, we evaluated two of our cell lines (UM-SCC-22a and UM-SCC-17b) with similar mutations (Fig. 8a, b). Compared with wild-type cells, these cell lines exhibited profoundly higher levels of acetylation of several histone marks, as well as increased global protein acetylation (Fig. 8a). Additionally, CBP protein itself was acetylated at higher levels compared with wild-type cells. As expected, the HAT inhibitor A-485 inhibited the observed acetylation in both mutant cell lines (Fig. 8b).

We then forced *CREBBP* wild-type (293T, HN31, and Detroit 562) cells to express full-length or a representative truncated inhibitory-region mutant (Q1773X). In all three lines, expression of the mutant led to a profound increase in CBP autoacetylation and global protein lysine acetylation that was not observed following forced expression of full-length CBP (Fig. 8c). Similarly, forced expression of mutant CBP led to increased acetylation at H3K18 and H3K27 histone marks (Fig. 8d). Additionally, we specifically examined the effects of mutant CBP on BRCA1 acetylation, which was similarly increased following forced expression of mutant CBP in HN31 and FaDu cell lines compared with wild type (Fig. 8e, f). Additionally, we examined the effects of forced expression of mutant CBP in both HN31 and FaDu cell lines on HR (Fig. 8g). In both cell lines, forced expression of mutant—but not wild-type—CBP led to significantly increased HR, which was abrogated by A-485 treatment.

### Mutations in *CREBBP*/*EP300* are associated with outcome following radiation in SCC

**Fig. 9:**
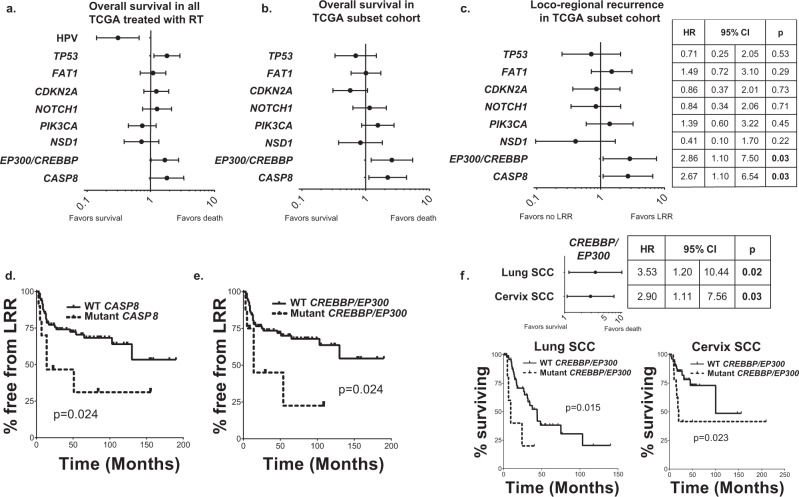
Whole-exome sequencing identifies *CREBBP*/*EP300* mutation significantly associated with outcome and treatment failure in several SCC cohorts. **a** Forrest plot of hazard ratios (HR) for overall survival (OS) for TCGA patients known to have received radiation therapy (*n* = 276 patients). **b**, **c** Forest plot of HR for OS (**b**) and locoregional recurrence (LRR) (**c**) in a subset of TCGA patients treated uniformly with surgery and radiation and known patterns of failure (*n* = 94 patients). **d**, **e** Kaplan–Meier curves for LRR in patients with either *CASP8* (**d**) or *CREBBP*/*EP300* (**e**) mutations. **f** Forest plot and KM curves for OS by *CREBBP*/*EP300* mutation in lung and cervix SCC cohorts.

To examine the clinical association between *CREBBP*/*EP300* mutation—and other mutations in HNSCC—with radioresponse, we identified a cohort of patients within the Head and Neck Cancer Genome Atlas (TCGA) that were denoted as having radiation as a component of their therapy (Supplementary Table 4). Genes significantly mutated at ≥10% frequency (high enough for potential clinical utility) were then queried to determine their relationship to overall survival (Fig. 9a). Only three genes (*TP53* (*p* = 0.015), *CASP8* (*p* = 0.055), and *CREBBP*/*EP300* (*p* = 0.046)) were associated with OS, as was the presence of HPV (*p* = 0.002) (47 patients, 17.4%) (Fig. 9a). Tumor stage (*p* = 0.69), nodal stage (*p* = 0.91), and tumor site (*p* = 0.79) were not significantly associated with survival in this population, this is likely due to the similar clinical characteristics of the selected cohort, all of whom had advanced-stage disease treated with combined modality therapy. While *TP53* was associated with OS in patients who did not receive radiation (HR 1.52, *p* = 0.051), *CASP8* (*p* = 0.41) and *CREBBP*/*EP300* (*p* = 0.89) were not, indicating that in the latter genes, this phenomenon may be dependent upon radiation response.

Because the TCGA includes a cohort of patients with heterogeneous treatments and without details of patterns of failure (particularly the difference between LRR and DM), we examined patient outcomes in a more uniformly treated cohort of patients within this group, in which treatment and patterns of failure details were enumerated (Fig. 9b, c). This subset included 94 patients with HPV-negative HNSCC treated uniformly with surgery and postoperative radiation (clinical characteristics in Supplementary Table 5). In this analysis, *TP53* (as a binary variable), was not associated with OS or LRR (Fig. 9b, c). Mutations in both *CASP8* and *CREBBP*/*EP300* were associated with significantly reduced overall survival and higher rates of LRR in this patient population (Fig. 9b–e).

Since *CREBBP*/*EP300* are commonly mutated in squamous tumors^27^, we expanded our analysis to other SCCs to determine whether their mutation might also be associated with poor outcome in other SCC tumor types treated with radiation. Among the TCGA squamous tumors, both lung and cervix had sufficient numbers and treatment annotations for analysis (clinical characteristics in Supplementary Table 6). We found that *CREBBP*/*EP300* mutations were associated with poorer overall survival in radiation-treated patients with lung and cervix squamous tumors (Fig. 9f).

## Discussion

**Fig. 10:**
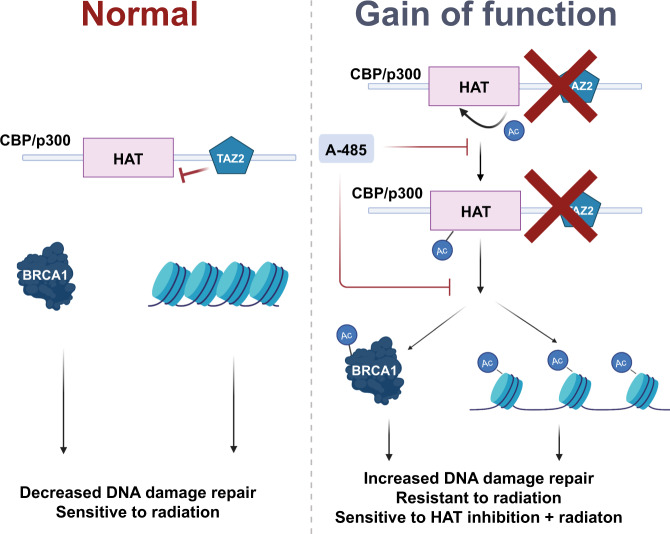
Proposed mechanism of *CREBBP*/*EP300* mutant-specific radiosensitization. Normal functioning of CBP and p300 shown on the left, with a potential gain of function (GOF) for both proteins shown on the right. The GOF mutation generally leads to increased protein acetylation, particularly BRCA1 acetylation, and increased DNA damage repair. Cells harboring these GOF mutations are sensitive to the combination of histone acetyltransferase (HAT) inhibition and radiation.

There are no biologically driven precision-medicine approaches to radiation therapy, with treatment largely guided by clinical stage and intensified via the addition of cytotoxic chemotherapy. This leads to high degrees of toxicity as well as both over- and undertreatment, depending upon the patient and tumor. HNSCC is no exception to this phenomenon, with a highly toxic standard treatment of concurrent chemoradiation that has largely remained unchanged for decades. To improve this paradigm, we performed in vivo screening of HNSCC models and identified both general radiosensitizing targets and a genomically associated sensitization. This latter effect is potentially related to a gain of function in mutant CBP and p300, leading to increased basal acetylation and BRCA1 function, rendering these tumors highly sensitive to the combination of HAT inhibition and radiation (potential mechanism in Fig. 10).

In this work, we performed an in vivo shRNA screen for targets associated with radiosensitization. We chose HNSCC as our initial model, both due to the primacy of radiation in curative therapy and the relative dearth of targetable genetic alterations in this malignancy. Importantly, the use of in vivo screening takes into account tumor bulk, metabolism, angiogenesis, and stromal interactions, which are not identified in in vitro screens. The identification of several genes identified as targets for clinical radiosensitization in HNSCC by our own group and others—notably *CHEK1*^28,29^, *PIK3CA*^15,30^, *PTK2*^16^, and *XIAP*^14^—argues for the utility of this technique in identifying more relevant targets for clinical trial development. Validation of additional general targets for radiosensitization identified in a similar manner is ongoing.

However, despite the potential for these targets to improve response, toxicity remains a concern for agents, such as PI3K^31,32^ or CHK1/2 inhibitors^33^, which exert broad antitumor effects when they are combined with DNA-damage-based therapies in the clinic. One means of partially mitigating the overlapping toxicities of radiation and targeted therapies is tailoring of specific agents to genomic events that drive radioresistance. Evaluating our screening data in this manner, we found that inhibiting the protein-acetylation function of CBP and p300 led to a dramatic sensitization to radiation in *CREBBP*/*EP300* mutant tumors and cell lines.

The concept of genomically driven radiation targeting is largely in its infancy. Despite ample evidence of genomic dependencies for targeted therapies—the classical example being PARP inhibition in *BRCA*-altered tumors^12^—direct links between a particular somatic mutation or genetic alteration and sensitivity to the combination of radiation and a particular agent are limited. Indeed, most studies of radiosensitizers have focused broadly on agents that either affect DDR or inhibit kinases known to be important in tumorigenesis. This approach has the advantage of a potentially broad applicability but risks masking effects in specific groups of patients, ultimately leading to underperforming clinical trials and/or unacceptable toxicities. Conversely, identifying a specific effect in *CREBBP*/*EP300* mutants, using a HAT inhibitor being developed for clinical use and radiation, can maximize response and tailor therapy to achieve a minimum of toxicity.

The identification of genomically associated radiosensitization in *CREBBP*/*EP300* mutants is particularly of interest in HNSCC, and indeed in SCCs in general, as we have shown in the current study that mutations in these genes are associated with clinical radioresistance. This is the first large-scale examination of somatic mutations in this context and serves to link particularly treatment-resistant tumors with a genomically tailored therapy.

*CREBBP* and *EP300* encode for homologous multifunctional bromodomain-containing acetyltransferases. Although these genes are mutated in HNSCC, they have not been extensively studied in this tumor type. Specifically, *CREBBP* and *EP300* are collectively mutated in 13% of HNSCC^34^, with similar frequencies in both HPV-positive and HPV-negative disease. Missense mutations are clustered in the acetyltransferase domain, and there is a reasonable frequency of truncating mutations (~20%). Additionally, many of these mutations are heterozygous, indicating possible haploinsufficiency, as has been seen for the chromatin-modifying genes in the BAF complex^35^. *CREBBP* and *EP300* are also mutated in 14% of all squamous cancers^27^, and we found them associated with poor survival in lung and cervical tumors (Fig. 9f). Although much of our work has been done in HNSCC, it should be applicable to other tumor types, particularly squamous-cell carcinoma. Furthermore, it is possible that the mechanism can be expanded to include other acetylation or chromatin-modifying genes and shed light on the role of acetylation in DNA-damage response and repair.

Wild-type CBP and p300 are both known to localize to sites of DNA damage, but their roles and putative interaction partners at these sites remain undefined. Both proteins have been implicated in nonhomologous end joining and homologous recombination, although to what extent this is a reflection of their ability to bind and modify repair proteins as opposed to modifying surrounding chromatin is unclear. For example, both were found to bind to acetylated E2F1 and this binding was required for recruitment of CBP and p300 to the sites of DNA damage^36^, but the authors attributed this interaction to CBP/p300 role in chromatin remodeling. Similarly, CBP/p300 were found to acetylate H3 and H4 at sites of double-strand breaks, a chromatin modification that was required for the binding of KU70/80 to damaged DNA^37^. Counterintuitively, in HeLa and 293T cells, p300 was found to interact with chromodomain helicase DNA-binding protein 4 (CHD4) in order to promote homologous recombination but not nonhomologous end joining^38^, suggesting that the role of CBP and p300 in specific DNA double-strand break repair pathways may be context dependent.

Lending further support to the idea that CBP/p300 play a role in homologous recombination, a recent report found that wild-type CBP/p300 promote transcription of BRCA1 and RAD51^39^. Using HeLa cells and two lung-cancer cell lines, the authors showed that CBP and p300 physically bind to the promoter regions of both these genes, but also affect acetylation of histones H3 and H4, which led to insufficient binding of transcription factors. From this work, it was unclear to what extent CBP and p300 actively participate in transcriptional control or if their involvement was due to regional histone modifications that enable binding of transcriptional factors. The same study also identified CBP and p300 as the critical factors promoting RPA loading to DNA ends following BRCA1 resection, further lending support to the likelihood that both proteins extend their participation in homologous recombination via modifications to the nearby chromatin.

Aside from chromatin modifications, CBP/p300 were also found to acetylate RAD52, leading to RAD52 localization to double-strand breaks and subsequent RAD51 accumulation and repair via homologous recombination^40^. This CBP/p300-mediated RAD52 modification was dependent on ATM, connecting the first DNA-damage response steps via ATM sensing to downstream repair-complex assembly via RAD52. This suggests that both CBP and p300 can directly modify DNA-repair proteins potentiating their functions, implying that in certain situations, CBP and p300 may play a role in cancer resistance to DNA-damaging therapy. What remains unclear is if these activities are actively selected for during tumor progression, enabling tumors to indirectly hyperactivate DNA-repair pathways.

Previously, one study has identified an “addiction” to p300 in the context of CBP deletion, which was felt to replicate naturally occurring mutations in *CREBBP*^41^. However, in our study, we did not observe an effect with p300 inhibition in *CREBBP* mutant cell lines in our model, either at baseline or in combination with radiation. We also did not observe the converse in *EP300* cell lines when CBP was inhibited. Thus, deletion of either protein may not recapitulate naturally occurring mutation.

Indeed, the data support a potentially more complex hypothesis of a gain of function for at least some mutations in *CREBBP*/*EP300*. Although we did not observe the effects on *BRCA1* transcription in either wild type or mutant cells following modulation of CBP, we did identify high basal levels of both protein—including BRCA1—and histone acetylation in cell lines harboring mutations, which truncate the protein downstream of the HAT domain, with forced expression of similar mutations in wild-type cell lines recapitulating this effect. This acetylation is reversed following inhibition of CBP and p300 HAT activity.

A gain of function for CBP and p300 is not wholly unprecedented, as previous data from the Cancer Cell Line Encyclopedia suggested a gain of function for certain *CREBBP* mutants, specifically truncation mutations located in the TAZ domain, for acetylation at certain histone marks, although this was only identified in a few cell lines^26^. However, based on our data, this gain of function—albeit with varying degrees of basal activity—may be more prevalent than previously appreciated. Moreover, this gain of function appears to extend to both increased autoacetylation and acetylation of additional proteins, notably BRCA1. This would be in addition to a basal increase in acetylation of histone tails (H3K18, H3K27, and H4K5/8/12/16), which serves to allow DDR proteins to access damaged DNA and facilitate its repair more easily^36,42^. We believe that this state functions to generally promote a state primed to repair DNA via increased BRCA1 activity and HR, compared with cell lines lacking these mutations. This state, in turn, renders the combination of HAT inhibition and radiation highly lethal, via an inhibition of HR (see Fig. 10).

This study is limited in that the full spectrum of mutations in *CREBBP* and *EP300* has not been studied. It is possible that the observed sensitization could be due to a combination of factors related to both a gain and loss of function for these proteins, particularly as the effect of missense mutations in the HAT domain, which are a significant proportion of all *CREBBP*/*EP300* mutations, is unclear. Similarly, we are not certain about the most appropriate terminology to use for this phenomenon. Our screen was analyzed to identify genomically associated sensitization, with several targets identified. However, the relationship between *CREBBP*/*EP300* mutations and genomically dependent targeting data is something akin to context-dependent oncogene addiction. However, many *CREBBP*/*EP300* mutations have patterns consistent with loss of function, so more mechanistic studies are necessary to clarify the phenotype. Additionally, although we have identified BRCA1 and HR as likely mediators of this phenomenon, because of the relatively broad effects of the examined mutations on protein acetylation, additional studies are needed to determine if additional signaling pathways modulate this effect.

In conclusion, we have both identified prognostic markers of outcome following radiation in SCC and explored radiosensitization involving one of these biomarkers, mutated *CREBBP*/*EP300*. This genomically associated radiosensitization appears to specifically involve effects on DNA-damage repair, leading to a HR deficiency following DNA damage and leading to increased apoptosis. A gain-of-function effect in mutated cell lines may be driving this phenomenon, leading to a basal hyperacetylated state affecting BRCA1 function, which is abrogated using a HAT inhibitor. This agent is currently being explored for clinical trial use, and thus could be translated clinically to improve outcomes in SCC.

## Methods

### Cell lines and chemicals

HNSCC cell lines (UM-SCC-47, UM-SCC-22a, UM-SCC-25, UM-SCC-1, HN31, HN30, UM-SCC-17B, UPCI:SCC-152, UD-SCC-2, Cal-27, and HN5) used in this study were generously supplied by Dr. Jeffrey Myers via The University of Texas MD Anderson Cancer Center Head and Neck cell line repository. HEK-293T, NCI-H520, NCI-H2228, NCI-H358, A549, Calu-6, FaDu, and Detroit 562 were purchased from American Type Culture Collection (Manassas, VA). Cell lines were tested for mycoplasma and genotyped before experiments.

UM-SCC-47, Cal 27, and UM-SCC-25 were maintained in Dulbecco modified Eagle medium (Gibco, USA), supplemented with 10% fetal bovine serum, 1% penicillin/streptomycin, 1% sodium pyruvate, 1% nonessential amino acids, and 2% vitamins. HEK-293T, HN5, HN30, HN31, UM-SCC-1, UM-SCC-17B, and UM-SCC-22a were maintained in DMEM/F-12 50/50 medium supplemented with 10% fetal bovine serum and 1% penicillin/streptomycin. UPCI:SCC-152, Detroit 562, Calu-6, and FaDu were maintained in MEM medium supplemented with 10% fetal bovine serum, 1% nonessential amino acids, and 1% penicillin/streptomycin. UD-SCC-2, NCI-H520, and NCI-H2228 were maintained in RPMI 1640 medium with 10% fetal bovine serum and 1% penicillin/streptomycin. All cell lines were incubated at 37 °C and 5% CO2 atmosphere.

ICG-001 was purchased from Selleck Chemicals (Houston, TX). A-485 and A-486 were manufactured by the MD Anderson Institute for Applied Cancer Science based on published structure^23^.

### Clonogenic survival assay

Single cells were plated in 6-well plates overnight. The next day, cells were incubated with specified drugs before irradiating at the indicated doses. The cells formed colonies over a 10- to 14-day incubation period and colonies were fixed in a 0.25% crystal violet/methanol solution. Colonies containing more than 50 cells each were counted. Survival curves were generated using GraphPad Prism (v8.0).

### Immunoblot analysis

Cells were washed in PBS and scraped and collected in sufficient amount of whole-cell lysis buffer (20 mM HEPES, pH 7.9, 0.4 M NaCl, 0.1 mM EDTA, pH 8, 0.1 mM EGTA, pH 7, 1% Igepal, and 1X Halt protease-inhibitor cocktail and 1X Halt phosphatase-inhibitor cocktail) (Thermo Sci). The lysate was mixed by vortexing and sonicated for 2 min at 100 amplitude with a QSonica Q700 sonicator (Newton, CT). Lysates were centrifuged at 20817 × *g* for 15 min at 4 °C. The supernatant was transferred to a fresh vial and total protein contents were estimated by DC Protein Assay kit (BioRad) and equal amounts of proteins were resolved on 4–15% gradient (SDS)-polyacrylamide gel (Bio-Rad). The proteins were electrotransferred for 10 min onto polyvinylidene-difluoride (PVDF) membrane using Transblot Turbo device (Bio-Rad). After blocking with 5% nonfat powdered milk in Tris-buffered saline (TBS, 0.1 M, pH = 7.4), blots were incubated with primary antibody at 4 °C overnight. The following primary antibodies were used: p300 (NM11)(1:1000, sc-32244) from Santa Cruz Biotechnology (Santa Cruz, CA); Acetyl-Lysine (RM101) from Abcam (Cambridge, United Kingdom) (1:500, ab190479); β-actin (C4) from MilliporeSigma (Burlington, MA) (1:10000, #MAB1501); and CBP (D6C5, 1:2000, #7389), H3K9Ac (C5B11, 1:5000, #9649), H3K18Ac (D8Z5H, 1:5000, #13998), H3K27Ac (D5E4, 1:5000, #8173), total Histone 3 (D1H2, 1:5000, #4499), and Cleaved Caspase-3 (5A1E, 1:500, #9664) from Cell Signaling Technology (Danvers, MA). After each step, blots were washed three times with Tween (0.1%)–Tris-buffer saline (TBS-T). Goat anti-mouse (1:2000, #NA931V) and anti-rabbit (1:2000, #NA934V) secondary antibodies conjugated to horseradish peroxidase (GE Healthcare, Chicago, Illinois) were used, and the signal was generated with the ECL2 western blotting substrate (Pierce Biotechnology, Rockford, IL) on HyBlot CL autoradiographic film (Thomas Scientific, Swedesboro, NJ). Protein abundance of β-actin served as a control for protein loading in each lane.

### Immunoprecipitation

Cells were grown in 15-cm dishes until 75% confluency and lysed using 1 ml of Pierce IP lysis buffer (Thermo) containing protease and phosphatase inhibitors. Lysates were sonicated for 2 min at 100% amplitude and cell debris was removed by centrifugation at 20817 × g for 15 min. Protein quantification was estimated using DC protein assay kit. About 500 µg of each sample were immunoprecipitated with 5 µl CBP antibody CBP (D6C5, 1:2000, #7389) (Cell Signaling Tech, Danvers, MA), 25 µl of BRCA1 antibody (D-9, 1:200, sc-9654) (Santa Cruz, CA), or anti-acetyl lysine affinity beads (AAC04-beads, Cytoskeleton Inc.), and incubated overnight rotating at 4 °C. For CBP and BRCA1 IP, 50 µl of 100 mg/ml Protein A Sepharose beads (17-0780-01, GE Healthcare) were added and rotated at 4 °C for 2 h the following day. Three 1-ml washes with IP lysis buffer were used to isolate the precipitate and samples were boiled in 25 µl of 2X SDS-loading buffer for 7 min and loaded into 4–15% polyacrylamide gels (BioRad).

### In vivo shRNA screen

Each library was cloned into the pRSI17 vector (Cellecta) and packed into lentivirus particles. HNSCC cell lines were infected in vitro through spinfection with virus containing the library at a low MOI (~20% infected cells as measured by flow cytometry) in order to minimize superinfection of cells. Cells were selected with puromycin for at least two days and grown in vitro for <3 population doublings prior to injection of 4 million cells subcutaneously in nude mouse flank. An additional 2 million cells from the day of injection were collected as a frozen reference-cell pellet. Pilot studies were performed to (i) examine the frequency of tumor initiating cells (TIC) and determine whether the cell line could maintain shRNA-library complexity in vivo and (ii) identify the dose of radiation needed to achieve ~20% tumor reduction for each model by the conclusion of the experiment.

For the screening experiment itself (and all other in vivo experiments), nude mice were housed between 68 and 79 °F, with 30–70% relative humidity and a 12 h:12 h light:dark cycle. Xenografts were treated with 2 Gy/day of radiation once the tumor had reached approximately 100 mm^3^ to a total dose of 6–10 Gy, depending upon the model. Following treatment, the tumors were allowed to grow for approximately two weeks (volume ~500 mm^3^). DNA was isolated from tumor and reference cells, amplified, and sequenced on Illumina sequencers^43^.

Hairpin counts were normalized to counts per million (CPM) per sample to enable comparison across samples. For each sample, (log2) fold-change of each hairpin in the tumor was calculated compared with the level in the reference pellet. A hairpin summary measure per cell line was derived from the median of quantile-transformed log2 FC across replicates. Next, a modified version of the redundant siRNA-activity (RSA) algorithm^44^ was used to derive a gene-level summary measure per cell line. RSA attempts to provide a gene level summary estimate of the impact of knockout of the gene by calculating a stepwise hypergeometric test for each hairpin in a gene. Similar to GSEA, it is based on evidence from multiple hairpins of a gene showing an impact of cellular fitness. Our modifications were to ensure both that at least two hairpins were used when calculating the minimum *p*-value (in RSA) and that hairpins ranking above luciferase controls were not used when determining the minimum *p*-value. Quantile rank of luciferase-control barcodes was determined through evaluation across all experiments; on an average luciferase barcodes ranked >0.6 on the quantile-transformed log2fc scale, so hairpins with quantile-transformed log2fc > 0.6 were not used for the gene-level RSA score. Data displayed graphically using JMP Pro (v14) and GraphPad Prism (v8.0).

### TUNEL assay

Following experimental treatments, all cells were collected, including floating cells, and TUNEL staining was performed using the APO-DIRECT Kit (BD Pharmingen) according to the manufacturer’s protocol. Briefly, 500,000 cells were fixed in 1% paraformaldehyde on ice for 30 min. Cells were then washed in PBS and fixed in 70% ethanol overnight at −20C. Cells were washed twice with provided buffer and then stained with 50 µl of DNA-labeling solution at 37 °C for 45-60 min. Cells were then rinsed twice with provided buffer and resuspended in 300ul of rinse buffer. Cells were then analyzed by flow cytometry using the BD Accuri C6 flow cytometer (BD Biosciences) with 488-nm laser, 533/30 filter, and FL1 detector. In total, 10,000 events were measured per sample. Standard SSC and FSC gating were used to exclude debris. From the gated dot-plot display, additional gating was applied at the edge of the unstained cell population (~4 log) and any events to the right of this population were gated as positive apoptosis (~5 log). About 2 µg/ml puromycin 24 and 48 h, in addition to kit controls, was used as positive-control samples to assist in proper delineation (exemplar gating shown in Supplementary Fig. 8a).

### Cell cycle

About 24 h after irradiation, all cells were collected including floating cells. Cells were then pelleted by centrifugation at 300 × *g* for 5 min and washed with PBS twice, then fixed using 70% ethanol for at least 30 min at 4 °C or overnight at -20 °C. After fixation, cells were pelleted and washed once with PBS, then resuspended in propidium iodide 50 ug/ml (Sigma Aldrich), 100 ug/ml Rnase A (Sigma Aldrich) in PBS, and incubated at room temperature for 30 min. Samples were then analyzed by flow cytometry using the Accuri C6 flow cytometer (BD Biosciences). Standard SSC and FSC gating were used to exclude debris. Standard gate was further gated by FL2-H and FL2-A, and additionally a third gating was applied, FL2-H by width, to remove doublets. A histogram was generated from these events and cell cycle distribution was quantified using FCS Express v7 using one-cycle DNA-fit analysis. Exemplar gating shown in Supplementary Fig. 8b.

### RT-PCR

Cells were collected using RNA-extraction buffer and passed through a QIAshredder (Qiagen). Followed manufacturer’s guidelines for RNA spin-column purification (RNeasy kit, Qiagen). Treated for 15 min at room temperature using DNaseI kit (Qiagen). Eluted total RNA with RNase-free water and quantified with nanodrop. Reverse-transcribed 1 µg of total RNA into cDNA using iScript RT supermix (BioRad). PCR priming for 5 min 25 °C, RT 40 min 42 °C, inactivation 5 min 85 °C, and cool ∞ 8 °C using T100 Thermal Cycler (BioRad). About 50 ng of cDNA in triplicate was mixed with primers for BRCA1, *CREBBP*, or GAPDH (BioRad, PrimePCR) and iTaq Universal SYBR green supermix (BioRad). Samples were amplified and quantified using CFX Connect Real-Time PCR (BioRad) and analyzed using BioRad CFX Manager. Primers listed in Supplementary Table 7

### Histone extraction

Histone proteins were extracted from treated or untreated cells using a histone-extraction kit (Abcam) according to the manufacturer’s protocol. Briefly, cells were harvested, and the pellet was obtained by centrifugation at 10621 × *g* for 5 min at 4 °C. The cells were resuspended in 1X prelysis buffer and incubated at 4 °C for 10 min on a rotator and then centrifuged for one minute at 10621 × *g* at 4 °C. The cell pellet was resuspended in lysis buffer at a concentration of 200 µL/10^7^ cells and incubated on ice for 30 min, then centrifuged at 15294 × *g* for 5 min at 4 °C. The supernatant was collected and 300 µL of balance buffer-DTT was added per 1 mL supernatant. The quantity of protein extracted was measured with a DC protein assay kit (Bio-Rad, Hercules, CA, USA). About 2–4 µg of protein per sample was separated by western blot analysis as described previously.

### Immunofluorescence staining

Immunofluorescence was performed to measure quantitative differences in DNA-damage repair and response. Cells were cultivated on cover slips placed in 35-mm cell culture dishes. At specified time points after exposure to radiation (2 Gy), cells were fixed in 4% paraformaldehyde for 10 min at room temperature on a shaker, briefly washed in phosphate-buffered saline or PBS (BioRad), and placed in 70% ethanol at 4 °C overnight. Fixed cells were washed with PBS twice to remove ethanol and permeabilized with 0.1% IGEPAL (octylphenoxypolyethoxyethanol) for 20 min at room temperature on a shaker, followed by blocking in 2% bovine serum albumin (Sigma) for 60 min, and then incubated with anti-γH2AX (s139,1:200, #2577), and 53BP1 (1:200, #4937), both from Cell Signaling Tech, Danvers, MA or anti-BRCA1 primary antibody (D-9, 1:200,#2577, Santa Cruz Technology, Santa Cruz, CA) overnight at 4 °C. The next day, fixed cells were washed three times with PBS and incubated for 45 minutes in the dark in secondary anti-mouse antibody conjugated to FITC (1:600, #715-165-150, Jackson ImmunoResearch, West Grove, PA) to visualize γH2AX or BRCA1. Secondary anti-rabbit antibody conjugated to Cy3 (1:600, #711-165-152, Jackson ImmunoResearch, West Grove, PA) was used to visualize 53BP1. DNA was stained with 4′,6-diamidino-2-phenylindole (Sigma) at 1:1000 (1 µg/ml). Immunoreactions were visualized with an Olympus or Leica Microsystems microscope (Wetzlar, Germany), and foci were counted with Image J software (https://imagej.nih.gov/ij/).

### Plasmids, shRNA, and siRNA transfection

For siRNA, 1 million cells were transfected in 100 µl of reagent T (Kit T, Lonza) using Nucleofector 2b electroporator (Amaxa) program T-020, with 200nmol *CREBBP* siRNA (ON-TARGETplus, Horizon Discovery Biosciences). Human *CREBBP* siRNA: GCACAGCCGUUUACCAUGA. Mock transfection was electroporated in 100 µl of reagent T only. Cells were collected for mRNA and western blot analysis 48–64 h after transfection.

For shRNA, packaging cell line HEK-293T was cotransfected with 3 µg of MISSION shRNAs specific for the *CREBBP*, *EP300* gene, or control (Millipore/Sigma) and lentiviral vectors DR8.2 and VSVG (Addgene). Two and three days after transfection, virus-containing media was filtered through a 0.45-µm PVDF syringe filter and polybrene was added (5 µg/ml, Sigma). Target cells were transduced with virus for 4–6 h and were subjected to puromycin antibiotic selection. Pooled knockdown cells and counterpart shControl cells were assessed for CBP protein expression by immunoblotting. shRNA sequences are given as follows:

shRNA *CREBBP* 2# TRCN0000006486 GCTATCAGAATAGGTATCATT

3# TRCN0000006487GCGTTTACATAAACAAGGCAT

shRNA *EP300* #2 TRCN0000039884 5′-CCAGCCTCAAACTACAATAAA-3′

#4 TRCN0000039886 5′-CCCGGTGAACTCTCCTATAAT-3′

#5 TRCN0000039887 5′-CGAGTCTTCTTTCTGACTCAA-3′

### Site-directed mutagenesis

Site-directed mutagenesis was performed using 50 ng of KAT3A/CBP (*CREBBP*) (NM_004380) Human Tagged ORF Clone (OriGene) as the dsDNA template. This was carried out using the QuickChange II XL Site-Directed Mutagenesis Kit (Agilent Technologies) according to the manufacturer’s protocol with the following exception: One Shot™ Stbl3™ Chemically Competent E. coli (Invitrogen) was used for transformation rather than the XL10-Gold Ultracompetent Cells supplied with the kit because of the dependency on chloramphenicol selection already found in the full-length *CREBBP* vector. Mutagenic oligonucleotide primers were designed using Agilent QuickChange Primer Design program and purchased from Sigma-Aldrich with PAGE purification with the following sequences:

5′-tggatgcagcgctagatgctcagccgg-3′

5′-ccggctgagcatctagcgctgcatcca-3′

Following transformation, single colonies were selected on LB plates containing 34ug/mL chloramphenicol, expanded in LB broth containing 34ug/mL chloramphenicol overnight, and extracted with a QIAfilter midi kit (Qiagen). Sanger sequencing was utilized to confirm the presence of the desired mutation. Mutant plasmids were directly transfected into cell lines using GeneJet transfection reagent (SignaGen Labs) or packaged in 293T for lentiviral infection.

### HR/NHEJ repair assays

For GeneJet transfection, FaDu and HEK293T cells were plated into 10-cm dishes. At 70% confluency, cells were transfected (GeneJet, SignaGen) with 5 µg of pDRGFP (Addgene, Plasmid #26475) or pimEJ5GFP (Addgene, Plasmid 44026) and stably selected with 2 µg/ml puromycin for two weeks. Stably selected cells were plated at 600,000 in 60-mm dishes and incubated overnight. The next day, cells were treated with 1.5µM A-485/A-486, 10 µM ATMi (KU-55933), or 100 nM ATRi (BAY-1895344) for 24 h. The following day, cells were transfected with 3 µg of pCBASceI (Addgene, Plasmid 26477) and 0.6 µg of mCherry (Addgene, Plasmid 41583) and incubated with drugs for a total of 72 h. Flow cytometry was run using BD Accuri C6 Plus and standard SSC and FSC gating excluded debris. A dot-plot display of FL1 (gfp) by FL2 (rfp) was gated at the edge of negative-control groups DRGFP or EJ5GFP. Any events to the right and upward from this gate were considered positive for repair (exemplar gating shown in Supplementary Fig. 8c). Data were plotted using GraphPad Prism v8.

For electroporation, UMSCC22A, HN31, HN30, A549, H460, Calu6, H520, H2228 and H358 5 µg of pDRGFP (Addgene, Plasmid #26475), or pimEJ5GFP (Addgene, Plasmid 44026) were electroporated using Nucleofector 2b technology (Amaxa) (see Supplementary Table 8 for programs used for individual cell lines) and stably selected with 2 µg/ml puromycin for two weeks. Stably selected cells were electroporated with 6 µg of pCBASceI (Addgene, Plasmid 26477) and 2 µg of mCherry (Addgene, Plasmid 41583) and incubated in 1.5µM A-485/A-486, 10 µM ATMi (KU-55933), or 100 nM ATRi (BAY-1895344) for a total of 72 h. Flow cytometry was run using BD Accuri C6 Plus and standard SSC and FSC gating excluded debris. A dot-plot display of FL1 (gfp) by FL2 (rfp) was gated at the edge of negative-control groups DRGFP or EJ5GFP. Any events to the right and upward from this gate were considered positive for repair. Data were plotted using GraphPad Prism v8.

### Mouse xenograft model

In vivo studies were performed according to all relevant ethical regulations and following Institutional Care and Use Committee (IACUC) approval from both the University of Pittsburgh and The University of Texas MD Anderson Cancer Center depending on the individual experiment. Male athymic nude mice (6–8-week old, ENVIGO/HARLAN, USA) were randomly assigned to treatment groups for each cell line tested (UM-SCC-47, UM-SCC-22a, and Detroit 562). Tumor cells (2 × 10^6^ in 0.1 mL of serum-free medium) were injected subcutaneously in the right dorsal flank of each mouse. After palpable tumors had developed, tumor diameters were measured with digital calipers, and tumor volume was calculated as A × B2 × 0.5, where A represents the largest diameter and B the smallest diameter. When the tumor volumes reached ~150 mm^3^, tumors were irradiated with 16 Gy (2 Gy/day x 8 days) and tracked for approximately four weeks for tumor-growth delay experiments (*n* = ~7–10/group). At that time, the experiment was completed, and tumors harvested. For in vivo TUNEL assay, tumors were collected 8 h after the last radiation treatment (*n* = 5/group). Days to reach 500 mm^3^ (UM-SCC-47 and Detroit 562) or 350 mm^3^ (UM-SCC-22a) for each group were calculated for each tumor and averaged within each treatment group rounded to the nearest day of measurement. Tumor volume was assessed at collection and averaged between groups. Kaplan–Meier curves were generated to evaluate survival, and group comparisons were performed using log-rank statistics.

### In vivo TUNEL assay

Paraffin-embedded sections (4 µm) of UM-SCC-47 tumor xenografts were mounted on coated slides and sent to HistoWiz Inc. (histowiz.com) for TUNEL staining and quantification. TUNEL staining was performed using a standard operating procedure and fully automated workflow with Deadend colorimetric TUNEL system from Promega. After staining, sections were dehydrated and film coverslipped using a TissueTek-Prisma and Coverslipper (Sakura). Whole-slide scanning (40x) was performed on an Aperio AT2 (Leica Biosystems). Images were analyzed using Halo (version 2.3.2089.34) image analysis software from Indica Labs (Albuquerque, NM). Regions of interest were selected. TUNEL staining was segmented using the CytoNuclear algorithm. Total cell counts were thresholded into low-, medium-, and high-intensity staining bins.

### Clinical data

This study was approved via the appropriate Institutional Review Board where applicable, complied with all relevant regulations regarding the use of human study participants, and was conducted in accordance with the criteria set by the Declaration of Helsinki. Informed consent was obtained for all participants in TCGA. The initial patient cohort consisted of the Head and Neck TCGA group that satisfied the following criteria: (i) whole-exome sequencing data are available and (ii) were denoted in the TCGA records as having received radiation as part of their initial therapy (clinical characteristics in Supplementary Table 4). Of the 523 patients in the TCGA cohort, a total of 276 patients met these criteria. Whole-exome sequencing from these tumors was examined for genes with mutations in ≥10% of tumors and significance on MutSig with the following genes meeting these criteria: *TP53*, *FAT1*, *CDKN2A*, *NOTCH1*, *NSD1*, *CREBBP*, and *EP300* (combined due to significant homology), and *CASP8*^34,45^. A subset (*n* = 94) of patients from this cohort have known specific treatment characteristics and patterns of failure analysis (clinical characteristics in Supplementary Table 5). These patients were all treated with surgery and post-operative radiation with well-annotated outcomes, including loco-regional recurrence and distant metastasis. In addition to the above, all tumors in this subset cohort were HPV/p16-negative.

Two additional cohorts from TCGA, the lung SCC and cervical SCC cohorts, were also examined, with a total of 61 and 66 patients respectively annotated as having received or who did likely receive (based on clinical scenario) external beam radiation and examined for outcomes (clinical characteristics in Supplementary Table 6).

For all clinical data, overall survival was defined from time of diagnosis until death or the last follow-up. Time to locoregional recurrence (LRR) or distant metastasis (DM) was defined as time from diagnosis until either an event or the last follow-up. Univariate analysis was performed using Cox regression (SPSS v25). Kaplan–Meier curves were generated, and group comparisons were performed using log-rank statistics.

### Statistics and reproducibility

Comparisons between groups were performed using ANOVA with post hoc analysis adjusted for multiple comparisons (Graphpad Prism v8). A minimum of three biologic replicates were used for all in vitro experiments and representative figures were repeated at least twice with similar results as shown. The UM-SCC-47 in vivo tumor-growth delay study was performed twice with similar results in both experiments. The remaining in vivo studies were performed once.

### Reporting summary

Further information on research design is available in the Nature Research Reporting Summary linked to this article.

## Supplementary information

Supplementary Information

Reporting Summary

## Data Availability

The data that support this study are available from the corresponding author upon reasonable request. Clinical outcome, tumor mutation, and gene expression data are publicly available from the Cancer Genome Atlas (TCGA) (https://gdac.broadinstitute.org/, https://www.cbioportal.org/study/summary?id=hnsc_tcga, and https://portal.gdc.cancer.gov/projects/TCGA-HNSC) (dbGaP Study Accession phs000178). All protocols used in this study are available in the Methods or Supplementary Methods sections. Source data for figures in the report are available as a separate excel file. Source data are provided with this paper.

## Source data

Source Data
