# Supplementary material for: Inhibition of histone acetyltransferase function radiosensitizes CREBBP/EP300 mutants via repression of homologous recombination, potentially targeting a gain of function
Source: Nat Commun. 2021 Nov 3;12:6340. doi: 10.1038/s41467-021-26570-8 (PMC8566594; doi:10.1038/s41467-021-26570-8)
Supplement: Supplementary file 1 — Supplementary Information [file 41467_2021_26570_MOESM1_ESM.pdf]

Supplementary Information for:

Inhibition of histone acetyltransferase function radiosensitizes CREBBP/EP300 mutants via repression of homologous recombination, potentially targeting a gain of function.

M. Kumar, D. Molkentine, J. Molkentine, K. Bridges, T. Xie, L. Yang, A. Hefner, M. Gao, R. Bahri, A. Dhawan, M. Frederick, S. Seth, M. Abdelhakiem, B. Beadle, F. Johnson, J. Wang, L. Shen, T. Heffernan, A. Sheth, R. Ferris, J. Myers, C. Pickering, H. Skinner.

Content of Supplementary Information:

Supplementary Table 1: Characteristics of the cancer cell lines used in this study.

Supplementary Table 2: Targets of the *in vivo* shRNA screening.

Supplementary Table 3: Targets from XRT vs. untreated tumor analysis.

Supplementary Table 3: Targets from XRT vs. untreated tumor analysis.

Supplementary Table 4: Clinical characteristics of the patients in the Head and Neck TCGA cohort treated with radiation.

Supplementary Table 5: Clinical characteristics of patients in the subset analysis.

Supplementary Table 6: Clinical characteristics of patients in the lung and cervix SCC cohorts.

Supplementary Table 7: RT-PCR primers used

Supplementary Table 8: Transfection protocols for individual cell lines.

Supplementary Figure 1: *In vivo* screening targets in CASP8 and NOTCH1 mutant tumors.

Supplementary Figure 2: Immunoblot for p300 in shRNA knockdown cells.

Supplementary Figure 3: Effects of *CREBBP* inhibition on DDR foci, cell cycle and BRCA1 transciption.

Supplementary Figure 4: Effects of ICG-001 on radioresponse in HNSCC cell lines.

Supplementary Figure 5: Clonogenic survival data following treatment with GNE-272, a bromodomain specific inhibitor for both CBP and p300.

Supplementary Figure 6: Effects of HAT inhibition in lung cancer cell lines.

Supplementary Figure 7: Baseline *CREBBP* and *EP300* expression.

Supplementary Figure 8: Gating strategy for flow cytometry experiments.

Supplementary Tables

Cell Line	CREBBP status	EP300 status	P53 status	HPV status
**Cal 27**	T2390N	WT	H193L	-
**UM-SCC-22a**	Q1773X	WT	Y220C, Splice site	-
**UM-SCC-17b**	S1761X	WT	R273C	-
**UM-SCC-25**	WT	P1780fs	Splice site	-
**HN5**	Q2158P	R1055X	C238S	-
**UM-SCC-47**	Q1092X	WT	WT	+
**HN31**	WT	WT	C176F, A161S	-
**HN30**	WT	Q793splice, V1594splice	WT	-
**UPCI:SCC152**	WT	WT	WT	+
**FaDu**	WT	WT	R248L, Splice site	-
**UM-SCC-1**	WT	Y1414C	Splice site	-
**Detroit 562**	WT	WT	R175H	-
**H520**	R1446C	WT	W146X	
**H2228**	WT	A2251T	Q331X	
**Calu6**	WT	WT	R196X	
**A549**	WT	WT	WT	
**H358**	WT	WT	Deep deletion	

Supplementary Table 1: Characteristics of the cancer cell lines used in this study.

**Targetable**				**DNA Damage**							
**ABL1**	FGFR4	MAPK7	PRKDC	ABCF2	CDK6	EXO1	MDM4	POLD3	REV3L	TCEA1	XRCC4
**ABL2**	FGR	MAPK8	PSMA1	ABL1	CDK7	FANCA	MGMT	POLDIP2	REXO2	TDG	XRCC5
**AKT1**	FLT1	MAPK9	PSMB1	AIFM1	CDKN1A	FANCB	MLH1	POLE	RFC1	TDP1	XRCC6
**AKT2**	FLT3	MAPT	PSMD1	ALKBH1	CDKN2A	FANCC	MLH3	POLE2	RFC2	TERF1	BP1
**AKT3**	FLT4	MCL1	PTCH1	ANKRD17	CDKN2B	FANCD2	MMS19	POLG	RFC3	TERF2	XRN2
**ALK**	FRK	MDM2	PTGS2	ANTXR1	CDKN2D	FANCE	MNAT1	POLG2	RFC4	TERT	YBX1
**AR**	FYN	MET	PTK2	APAF1	CDT1	FANCF	MPG	POLH	RFC5	TFF2	ZAK
**ATM**	GLS	MKNK1	PTPN11	APEX1	CEBPG	FANCG	MRE11A	POLI	RFWD2	TGFB1	ZW10
**ATR**	GSK3A	MS4A1	PTPN6	APEX2	CENPF	FANCI	MSH2	POLK	RINT1	TIMELESS	ZWINT
**AURKA**	GSK3B	MTOR	RAC1	APTX	CETN2	FANCL	MSH3	POLL	RNF168	TIPIN	
**AURKB**	HDAC1	MYC	RAF1	ASF1A	CHAF1A	FANCM	MSH4	POLM	RNF8	TNFRSF10B	
**AURKC**	HDAC2	NAMPT	RARA	ATM	CHAF1B	FEN1	MSH5	POLN	RPA1	TNP1	
**AXL**	HDAC3	NFKB1	RARB	ATR	CHEK1	FOXN3	MSH6	POLQ	RPA2	TOP1	
**BCL2**	HDAC6	NOTCH1	RARG	ATRIP	CHEK2	FUS	MTOR	POLR2G	RPA3	TOP2A	
**BCR**	HDAC8	NR2C2	RET	ATRX	CHFR	GADD45A	MUS81	POT1	RPA4	TOPBP1	
**BIRC5**	HSP90AA1	NTRK1	ROCK1	ATXN3	CIB1	GADD45B	MUTYH	PPM1D	RPAIN	TP53	
**BLK**	IDH1	NUDT1	ROCK2	BAI1	CIDEA	GADD45G	NAE1	PRIM1	RPL13A	TP53BP1	
**BMX**	IDH2	P4HB	RPL30	BARD1	CRY1	GTF2E2	NBN	PRIM2	RPL30	TP73	
**BRAF**	IGF1R	PAK1	RPS6KB1	BCL2	CRY2	GTF2H1	NCOA6	PRKCG	RPRM	TREX1	
**BRD4**	IKBKE	PAK4	RRM1	BIRC5	CSNK1D	GTF2H2	NEIL1	PRKDC	RPS27A	TREX2	
**BTK**	IL1B	PARP1	RXRA	BLM	CSNK1E	GTF2H2B	NEIL2	PSMA1	RRM1	TRIAP1	
**CASP3**	IL6	PARP2	RXRB	BRCA1	CUL4A	GTF2H3	NEK11	PSME4	RRM2	TRRAP	
**CCR5**	IL6R	PARP3	SGK3	BRCA2	CUL4B	GTF2H4	NHEJ1	PTEN	RRM2B	TTK	
**CD274**	INSR	PDGFRA	SMO	BRIP1	CYCS	GTF2H5	NME2	PTTG1	RUVBL1	TXN	
**CD52**	IRAK4	PDGFRB	SRC	BRSK1	DCLRE1A	GTSE1	NTHL1	RAD1	RUVBL2	UBA52	
**CDK1**	ITK	PDK1	STAT3	BTG2	DCLRE1B	H2AFX	NUDT1	RAD17	SEMA4A	UBB	
**CDK2**	JAK1	PGD	SYK	BUB1	DCLRE1C	HDAC4	OGG1	RAD18	SESN1	UBE2A	
**CDK4**	JAK2	PIGF	TBK1	BUB1B	DDB1	HMGB2	OXR1	RAD21	SETX	UBE2B	
**CDK6**	JAK3	PIK3CA	TEC	CCNA2	DDB2	HPRT1	PALB2	RAD23A	SF3B3	UBE2I	
**CDK7**	KDM1A	PIK3CB	TEK	CCNB1	DDIT3	HUS1	PARG	RAD23B	SHFM1	UBE2N	
**CDK9**	KDR	PIK3CD	TNF	CCNB2	DDX11	IGHMBP2	PARP1	RAD50	SHISA5	UBE2T	
**CHEK1**	KIT	PIK3CG	TNFRSF8	CCNB3	DKC1	ING1	PARP2	RAD51	SIAH1	UBE2V1	
**CHEK2**	LAP3	PIM1	TNFSF11	CCND1	DLGAP5	INPPL1	PARP3	RAD51B	SIRT1	UBE2V2	
**CREBBP**	LCK	PIM2	TNFSF13B	CCND2	DMC1	IP6K3	PARP4	RAD51C	SLK	UNG	
**CTNNB1**	LDHA	PIM3	TOP1	CCND3	DNA2	KAT2A	PCNA	RAD51D	SLX4	UPF1	
**DHFR**	LUC	PLK1	TOP2A	CCNE1	DNTT	KAT5	PERP	RAD52	SMC1A	USP1	
**DOT1L**	LYN	PORCN	TOP2B	CCNE2	DUT	KNTC1	PML	RAD54B	SMC2	UVRAG	
**DRD2**	MAP2	PPM1D	TRIM24	CCNG1	E2F1	LIG1	PMS1	RAD54L	SMC3	VCP	
**EGFR**	MAP2K1	PRKAA1	TRPV1	CCNG2	EI24	LIG3	PMS2	RAD9A	SMC6	WDR33	
**EHMT2**	MAP2K2	PRKCA	TUBB	CCNH	EME1	LIG4	PMS2L2	RB1	SOD1	WRAP53	
**EIF4E**	MAP3K14	PRKCB	TXN	CCNO	EP300	LRIG1	PMS2P1	RBBP4	SPO11	WRN	
**EPHA2**	MAP3K8	PRKCD	TYMS	CDC25A	ERCC1	LUC	PMS2P3	RBBP8	SSBP1	WRNIP1	
**ERBB2**	MAP4	PRKCE	TYRO3	CDC25B	ERCC2	MAD2L1	PMS2P4	RBM14	STEAP3	XAB2	
**ESR1**	MAPK1	PRKCG	VEGFA	CDC25C	ERCC3	MAD2L2	PMS2P5	RECQL	SUMO1	XPA	
**ESR2**	MAPK11	PRKCH	WEE1	CDC6	ERCC4	MBD4	PNKP	RECQL4	SUPT3H	XPC	
**EZH2**	MAPK12	PRKCI	WHSC1	CDK1	ERCC5	MCM8	POLA1	RECQL5	TADA3	XRCC1	
**FGFR1**	MAPK13	PRKCQ	XIAP	CDK2	ERCC6	MDC1	POLB	RELA	TAF2	XRCC2	
**FGFR2**	MAPK14	PRKCSH	XPO1	CDK4	ERCC8	MDM2	POLD1	REV1	TAF5L	XRCC3	
**FGFR3**	MAPK3	PRKCZ									

Supplementary Table 2: Targets of the *in vivo* shRNA screening.

Gene	Average untreated	Average XRT	Gene	Average untreated	Average XRT	Gene	Average untreated	Average XRT
ABL1	-0.549	-0.858	*KDM1A*	-0.666	-1.080	*RUVBL1*	-1.964	-2.683
AKT1	-0.720	-1.222	*LCK*	-0.917	-1.401	*SEMA4A*	-0.626	-0.947
ANTXR1	-0.909	-1.230	*MAD2L1*	-1.303	-1.534	*SF3B3*	-1.522	-1.704
ATM	-1.122	-1.278	*MAP3K8*	-1.906	-2.190	*SMC1A*	-2.111	-2.918
ATRIP	-1.806	-2.370	*MAPK12*	-0.653	-1.095	*SMC6*	-2.018	-2.506
ATXN3	-0.700	-0.945	*MCL1*	-1.321	-1.708	*SPO11*	-0.683	-1.254
AURKB	-0.900	-1.042	*MDM2*	-0.691	-1.165	*SSBP1*	-0.946	-1.442
BCL2	-0.629	-1.051	*MKNK1*	-0.553	-0.901	*SYK*	-0.742	-0.924
BLK	-0.864	-1.115	*MSH5*	-0.513	-1.032	*TOP2A*	-0.660	-0.976
BLM	-0.607	-0.896	*MTOR*	-0.774	-0.949	*TOPBP1*	-0.816	-1.007
BRAF	-1.084	-1.349	*MTOR*	-0.934	-1.337	*TP53BP1*	-0.674	-1.477
BRCA2	-0.600	-1.059	*NEIL2*	-1.800	-2.093	*TTK*	-0.515	-0.954
BRD4	-1.228	-1.553	*NOTCH1*	-0.980	-1.167	*TXN*	-0.747	-1.029
BTG2	-0.964	-1.090	*NUDT1*	-1.261	-1.598	*TYMS*	-0.743	-0.934
BTK	-0.636	-0.945	*OXR1*	-0.756	-0.867	*UBB*	-0.750	-0.900
CCNB1	-1.136	-1.300	*PAK4*	-0.916	-1.113	*UBE2A*	-0.670	-0.911
CCNO	-1.803	-2.782	*PARG*	-1.568	-1.961	*UBE2V2*	-2.217	-2.590
CDC25A	-1.057	-1.372	*PARP4*	-0.679	-1.002	*VCP*	-1.497	-1.647
CDC25B	-0.713	-0.867	*PERP*	-0.296	-0.814	*XIAP*	-1.729	-2.352
CDK2	-0.843	-1.069	*PIGF*	-0.769	-0.894	*XPA*	-0.645	-0.863
CDK4	-0.958	-1.315	*PIK3CA*	-2.798	-3.597	*XRCC5*	-1.064	-1.238
CENPF	-0.781	-1.071	*PMS1*	-0.948	-1.179	*XRCC6*	-1.431	-1.732
CHEK1	-1.352	-1.741	*PMS2P3*	-0.357	-0.904	*YBX1*	-1.912	-2.264
CIB1	-0.803	-0.942	*PMS2P5*	-0.765	-1.282	*ZW10*	-0.977	-1.289
CREBBP	-0.588	-0.899	*POLA1*	-1.097	-1.618	*ZWINT*	-0.963	-1.829
CUL4A	-0.670	-1.144	*POLG*	-1.016	-1.311			
DLGAP5	-0.810	-0.971	*POLL*	-1.115	-1.416			
DRD2	-0.608	-1.037	*PRKCQ*	-0.619	-0.857			
DUT	-1.763	-2.038	*PRKDC*	-0.942	-1.266			
EHMT2	-0.688	-1.015	*PRKDC*	-1.508	-1.807			
EP300	-0.589	-1.010	*PTK2*	-1.545	-1.857			
EPHA2	-1.264	-1.537	*RAD1*	-0.805	-1.076			
ERCC6	-1.241	-1.389	*RAD51*	-1.151	-1.443			
ERCC8	-0.962	-1.179	*RAD51C*	-0.797	-0.960			
ESR1	-0.817	-1.396	*REXO2*	-0.907	-1.118			
FANCD2	-0.727	-1.157	*RFC2*	-0.881	-1.000			
FANCE	-0.840	-1.435	*RNF168*	-0.866	-1.118			
FANCL	-0.973	-1.144	*RPA1*	-1.149	-1.277			
FGFR4	-1.163	-1.410	*RPA2*	-2.374	-2.761			
GTF2E2	-1.113	-1.232	*RPRM*	-0.587	-0.989			
JAK2	-0.693	-1.071	*RPS6KB1*	-0.663	-0.969			
KAT2A	-1.173	-1.353	*RRM2B*	-0.827	-0.927			

Supplementary Table 3: Targets from XRT vs. untreated tumor analysis.

Site	N	%
Oral Cavity	152	55.1%
Oropharynx	56	20.3%
Larynx/hypopharynx	68	24.6%
Nodal stage		
0	101	36.9%
1	53	19.3%
2x	3	1.1%
2a	14	5.1%
2b	61	22.3%
2c	29	10.6%
3	5	1.8%
Unknown	8	2.9%
Tumor stage		
1	15	5.5%
2	60	21.9%
3	78	28.5%
4	115	42.0%
Unknown	6	2.2%

Supplementary Table 4: Clinical characteristics of the patients in the Head and Neck TCGA cohort treated with radiation.

Site	N	%
Oral Cavity	60	63.8%
Oropharynx	4	4.3%
Larynx/hypopharynx	30	31.9%
Nodal stage		
1	44	46.8%
2x	20	21.3%
2a	1	1.1%
2b	2	2.1%
2c	13	13.8%
3	8	8.5%
Unknown	2	2.1%
	4	4.3%
Tumor stage		
1	1	1.1%
2	12	12.8%
3	23	24.5%
4	56	59.6%
Unknown	2	2.1%

Supplementary Table 5: Clinical characteristics of patients in the subset analysis.

	Lung SCC				Cervix SCC	
**Overall stage**	**N**	**%**		**Overall stage**	**N**	**%**
**IA**	2	3.3		IB1	10	15.2
**IB**	4	6.6		IB2	6	9.1
**II**	1	1.6		IIA	2	3.0
**IIA**	6	9.8		IIA2	1	1.5
**IIB**	14	23.0		IIB	22	33.3
**III**	1	1.6		III	1	1.5
**IIIA**	26	42.6		IIIA	3	4.5
**IIIB**	7	11.5		IIIB	11	16.7
				IVA	3	4.5
**Nodal stage**				IVB	7	10.6
**N0**	16	26.2				
**N1**	17	27.9		**Nodal stage**		
**N2**	22	36.1		N0	11	16.7
**N3**	5	8.2		N1	8	12.1
**NX**	1	1.6		NX	47	71.2
**Tumor stage**				**Tumor stage**		
**T1**	4	6.6		T1b	2	3.0
**T2**	24	39.3		T1b1	6	9.1
**T2a**	6	9.8		T1b2	4	6.1
**T2b**	4	6.6		T2	2	3.0
**T3**	18	29.5		T2a	2	3.0
**T4**	5	8.2		T2a2	2	3.0
				T2b	18	27.3
				T3	2	3.0
				T3a	2	3.0
				T3b	10	15.2
				T4	5	7.6
				Tis	1	1.5
				TX	10	15.2
				**Grade**		
				G1	3	4.5
				G2	28	42.4
				G3	23	34.8
				GX	12	18.2

Supplementary Table 6: Clinical characteristics of patients in the lung and cervix SCC cohorts.

**RT-PCR primers**				
**PrimePCR Assay (BioRad)**				
**Gene**	**Cat#**	**Species**	**Tag**	**Unique assay ID**
CREBBP	10025636	human	Sybr Green	qHsaCID0021913
BRCA1	10025636	human	Sybr Green	qHsaCED0038604
GAPDH	10025636	human	Sybr Green	qHsaCED0038674

Supplementary Table 7: RT-PCR primers used

Cells	Kit	Program	Cells	Efficiency	Viable Cells	Substrate		mCherry	Format	Platform
**A549**	T	T-020	1.00E+06	70%	80%	Plasmid (general)	6 μg	2ug	100 µl	I/II/2b
**Calu6**	L	X-005	2.00E+06	75%	50%	Plasmid (general)	6 μg	2ug	100 µl	I/II/2b
**H2228**	T	X-001	3.00E+06	25%	50%	Plasmid (general)	6 μg	2ug	100 µl	I/II/2b
**H358**	T	A-023	1.00E+06	80%	75%	Plasmid (general)	6 μg	2ug	100 µl	I/II/2b
**H441**	T	A-020	2.00E+06	75%	50%	Plasmid (general)	6 μg	2ug	100 µl	I/II/2b
**H520**	T	T-020	3.00E+06	33%	25%	Plasmid (general)	6 μg	2ug	100 µl	I/II/2b
**H460**	T	T-020	1.50E+06	25%	75%	Plasmid (general)	6 μg	2ug	100 µl	I/II/2b
**FaDu**				80%	95%	Plasmid (general)	3 μg	0.6ug	9 µl	GenJet
**293T**				95%	100%	Plasmid (general)	3 μg	0.6ug	9 µl	GenJet
**UM-SCC-22a**	T	T-020	1.00E+06	50%	50%	Plasmid (general)	6 μg	2ug	100 µl	I/II/2b
**HN31**	T	T-020	1.00E+06	75%	40%	Plasmid (general)	6 μg	2ug	100 µl	I/II/2b
**HN30**	T	T-020	2.00E+06	40%	40%	Plasmid (general)	6 μg	2ug	100 µl	I/II/2b

Supplementary Table 8: Transfection protocols for individual cell lines.

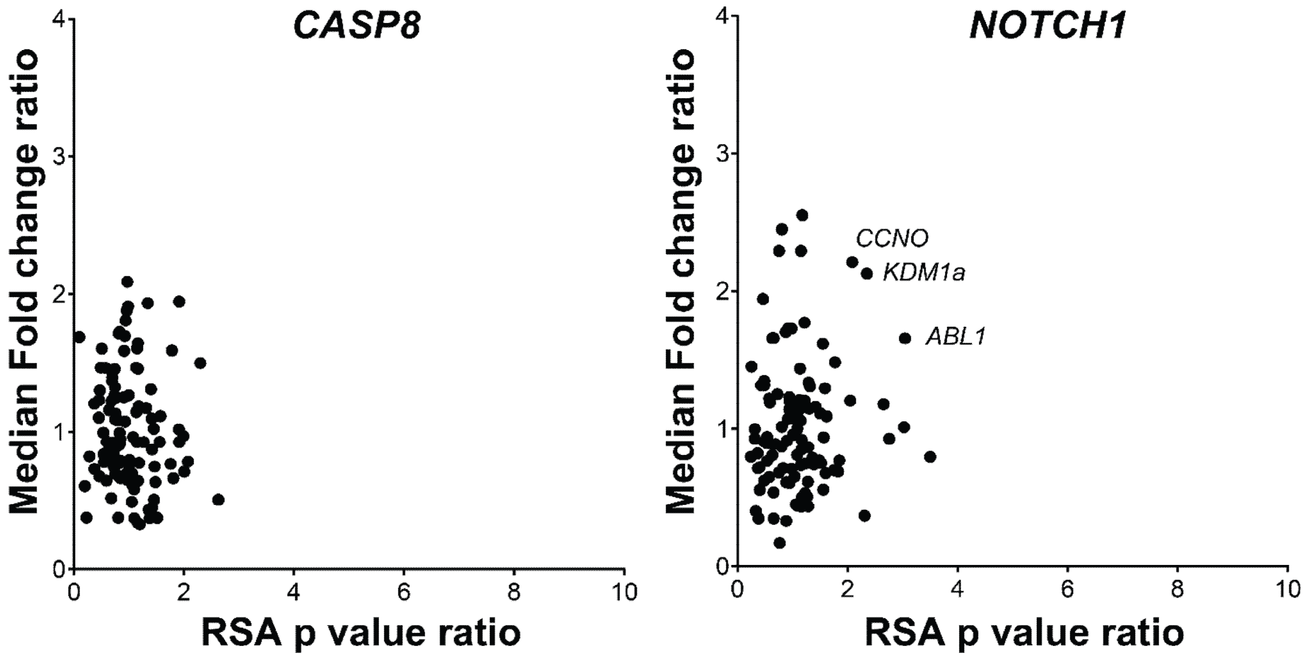

Supplementary Figure 1: *In vivo* screening targets in CASP8 and NOTCH1 mutant tumors. Ratio of CASP8 or NOTCH1 mutant vs. wild type for target fold change (y-axis) and RSA log p-value (x-axis) for radiosensitizing targets selected from Fig. 1.

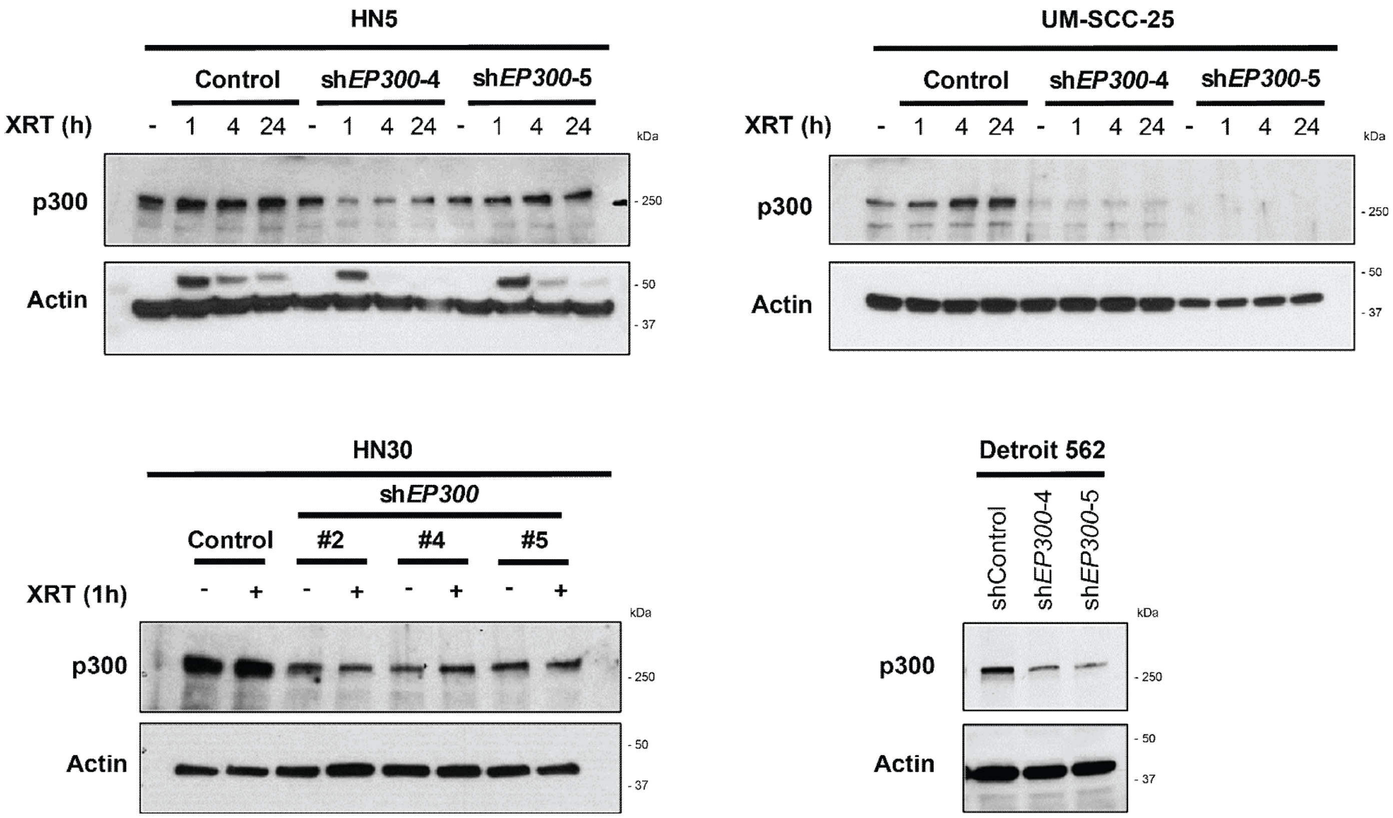

Supplementary Figure 2: Immunoblot for p300 in shRNA knockdown cells.

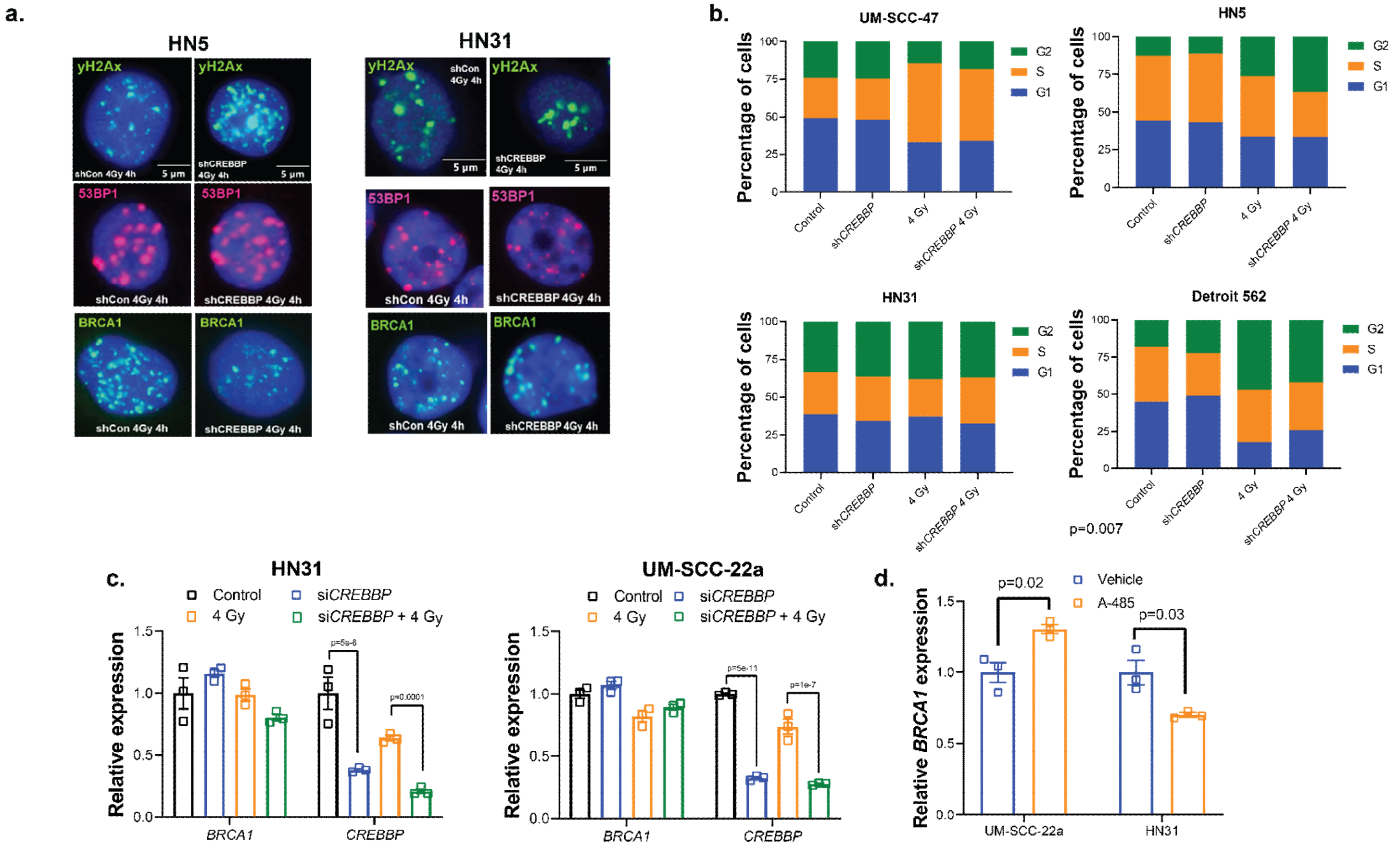

Supplementary Figure 3: Effects of *CREBBP* inhibition on DDR foci, cell cycle and BRCA1 transciption. a) Representative foci staining from *CREBBP/EP300* mutant (HN5) and wild type (HN31) cells. b) Cell cycle analysis in control and *CREBBP* knockdown cells at baseline and 24h following 4 Gy. c) *BRCA1* and *CREBBP* gene expression at baseline or 24h following 4 Gy. d) *BRCA1* transcription following 24h treatment with A-485. For c & d, a minimum of 3 independent samples for each condition are shown and are presented as mean values +/- SEM with two-sided p-values shown.

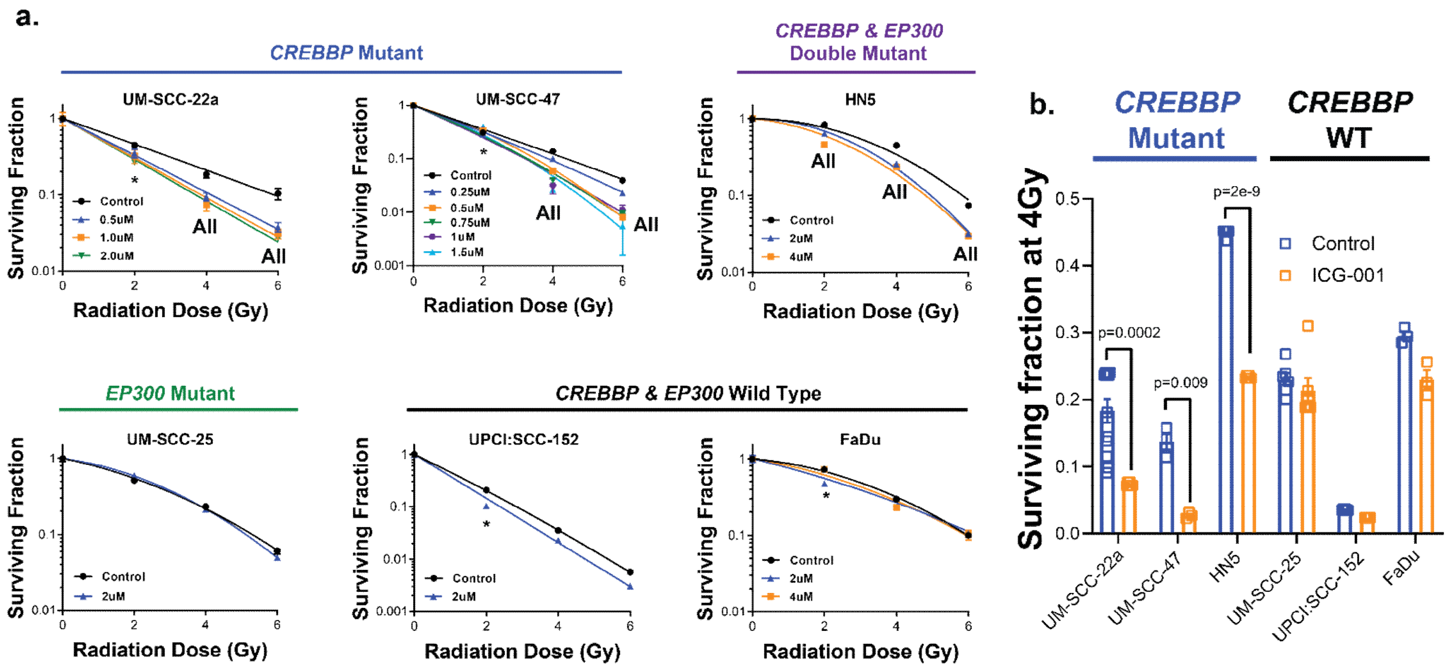

Supplementary Figure 4: Effects of ICG-001 on radioresponse in HNSCC cell lines. a) Clonogenic survival following irradiation and ICG-001 in *CREBBP* mutant and wild type cell lines. b) Surviving fraction at 4 Gy in cell lines from (a). Clonogenic survival curves analyzed as in Fig. 2. A minimum of 3 independent samples for each condition are shown and are presented as mean values +/- SEM. (All) – all ICG-001 doses two-sided $p<0.05$ versus control, otherwise two-sided p-value versus vehicle control as shown.

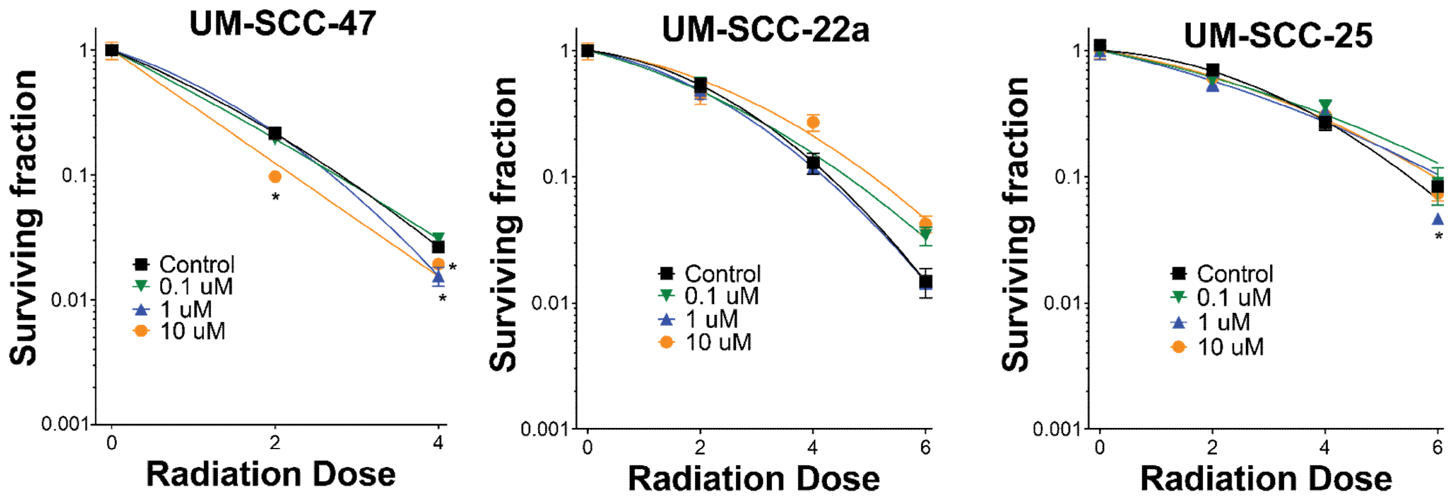

Supplementary Figure 5: Clonogenic survival data following treatment with GNE-272, a bromodomain specific inhibitor for both CBP and p300. Clonogenic survival curves analyzed as in Fig. 2. A minimum of 3 independent samples for each condition are shown and are presented as mean values +/- SEM. (*) – two-sided $p<0.05$ versus vehicle control.

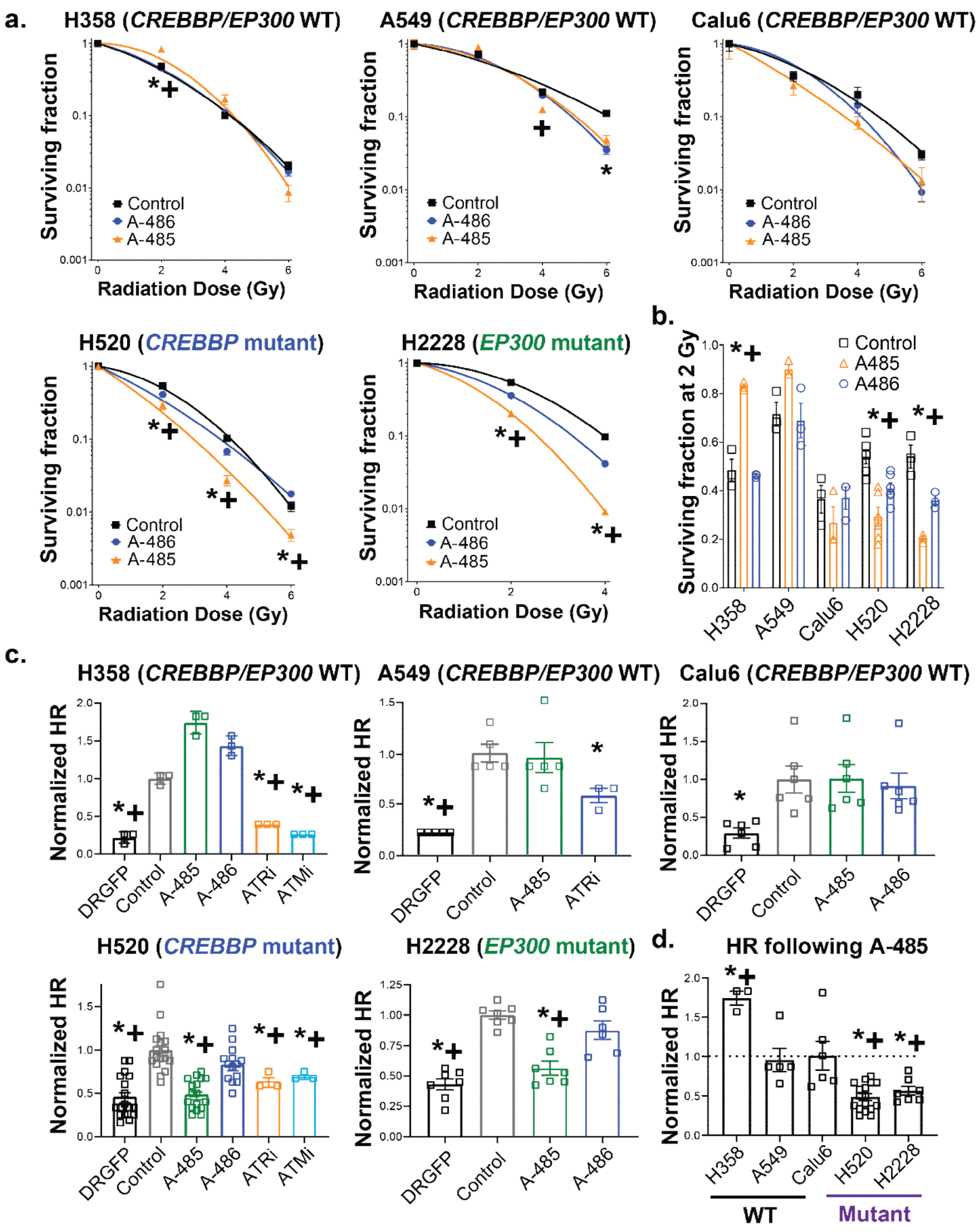

Supplementary Figure 6: Effects of HAT inhibition in lung cancer cell lines. a) Clonogenic survival following irradiation and either A-485 (active) or A-486 (inactive) in *CREBBP/EP300* mutant or wild type lung cancer cells. b) Surviving fraction at 2 Gy in cell lines from (a). c) I-SceI assay for HR as described in the methods following treatment with A-485, A-486 or inhibitors of either ATM or ATR, with normalized HR for each line after A-485 shown in (d). p-values are two-sided and derived from ANOVA with post-hoc analysis adjusted for multiple comparisons. A minimum of 3 independent samples for each condition are shown and are presented as mean values +/- SEM. (*,+) – p<0.05 vs. Control (*) and A-486 (+).

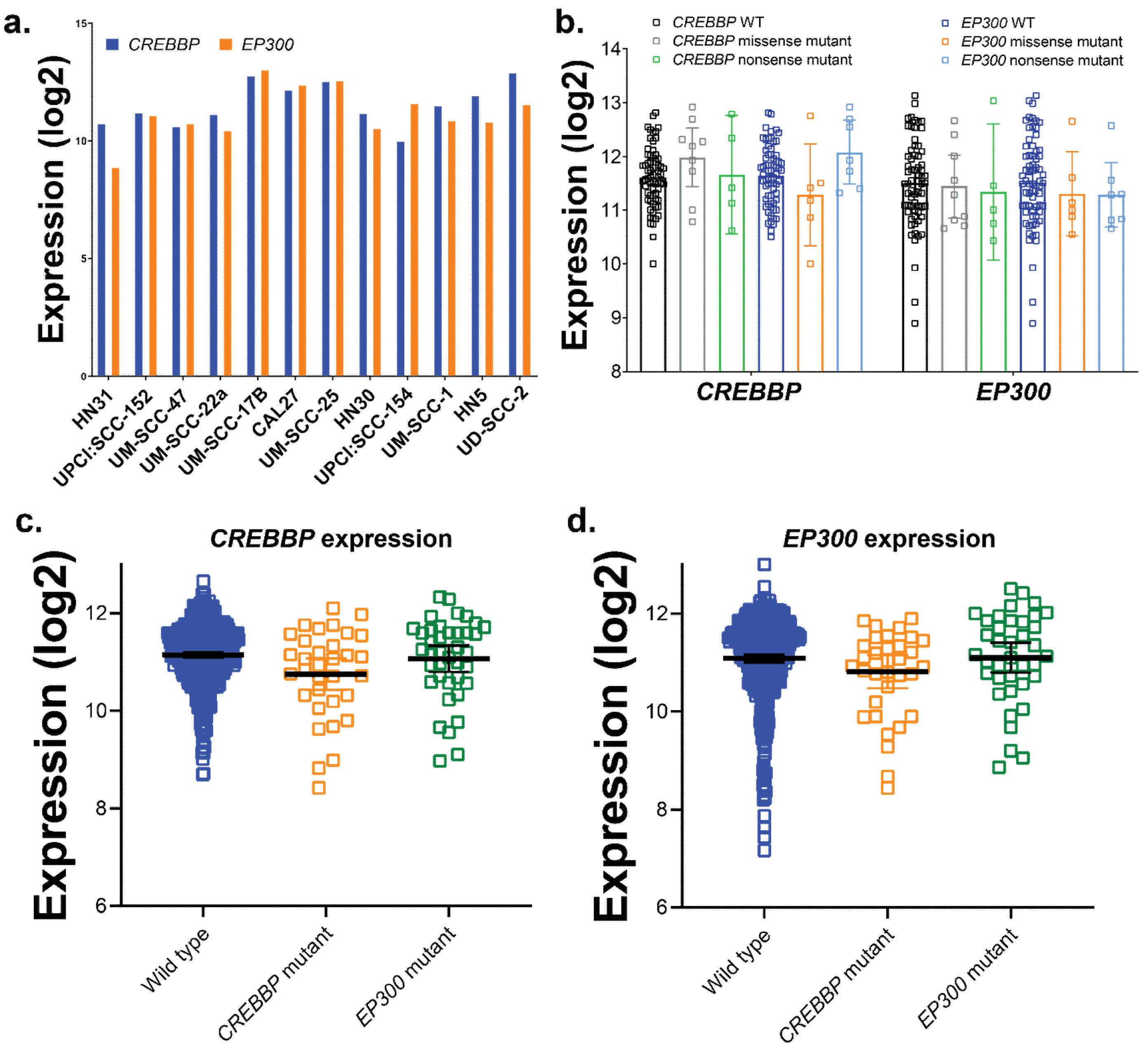

Supplementary Figure 7: Baseline *CREBBP* and *EP300* expression. a & b) *CREBBP* and *EP300* mRNA expression in the cell lines used in this study (a) and in a total of 82 HNSCC cell lines described previously (b). c & d) *CREBBP* (c) and *EP300* (d) mRNA expression of all available tumors from the Head and Neck TCGA (total n=522), with data are presented mean values +/- SEM.

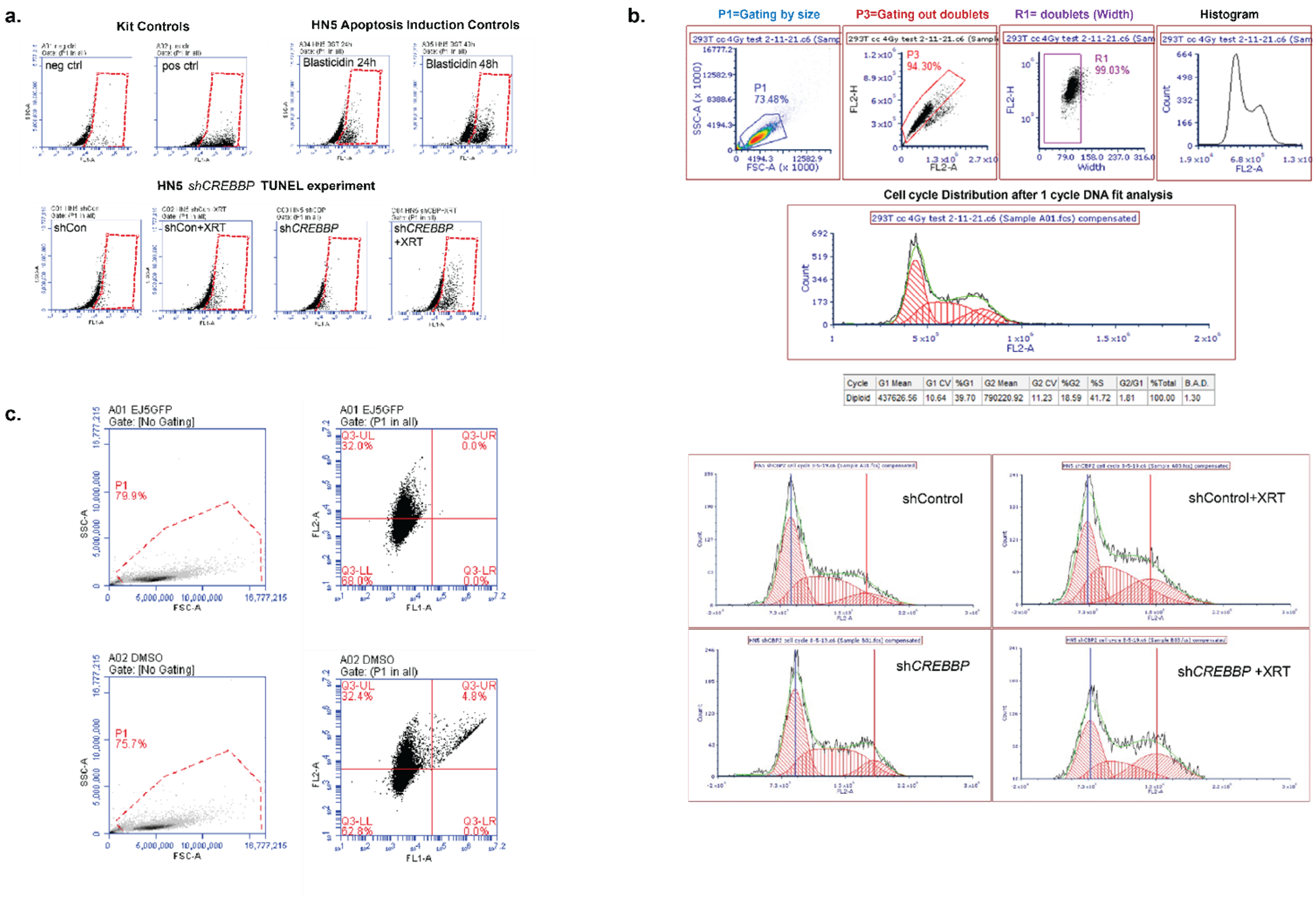

Supplementary Figure 8: Gating strategy for flow cytometry experiments. Detailed methods for flow experiments found in the Methods section of the main manuscript. a) Standard SSC and FSC gating were used to exclude debris, with 10,000 cells within the SSC/FCS gate further analyzed. From the gated dot plot display, additional gating was applied at the edge of the unstained cell population (~4 log) and any events to the right of this population was gated as positive apoptosis (~5 log). 2µg/ml puromycin 24 and 48h, in addition to kit controls, were used as positive control samples to assist in proper delineation. b) Standard SSC and FSC gating were used to exclude debris, with 10,000 cells within the SSC/FCS gate further analyzed. Standard gate was further gated by FL2-H and FL2-A, and additionally a third gating was applied, FL2-H by width, to remove doublets. A histogram was generated from these events and cell cycle distribution was quantified using FCS Express v7 using 1 cycle DNA fit analysis. c) Standard SSC and FSC gating excluded debris and 5000 cells within the SSC/FCS gate were further analyzed. A dot plot display of FL1 (gfp) by FL2 (rfp) were gated at the edge of negative control groups DRGFP or EJ5GFP. Any events to the right and upward from this gate were considered positive for repair.
