# Supplementary material for: Inhibition of histone acetyltransferase function radiosensitizes CREBBP/EP300 mutants via repression of homologous recombination, potentially targeting a gain of function
Source: Nat Commun. 2021 Nov 3;12:6340. doi: 10.1038/s41467-021-26570-8 (PMC8566594; doi:10.1038/s41467-021-26570-8)
Supplement: Supplementary file 2 — Reporting Summary [file 41467_2021_26570_MOESM2_ESM.pdf]

nature portfolio

Corresponding author(s): Heath Skinner

Last updated by author(s): Sep 20, 2021

Reporting Summary

Nature Portfolio wishes to improve the reproducibility of the work that we publish. This form provides structure for consistency and transparency in reporting. For further information on Nature Portfolio policies, see our Editorial Policies and the Editorial Policy Checklist.

Statistics

For all statistical analyses, confirm that the following items are present in the figure legend, table legend, main text, or Methods section.

n/a	Confirmed	
☐	☒	The exact sample size (*n*) for each experimental group/condition, given as a discrete number and unit of measurement
☒	☐	A statement on whether measurements were taken from distinct samples or whether the same sample was measured repeatedly
☐	☒	The statistical test(s) used AND whether they are one- or two-sided<br>*Only common tests should be described solely by name; describe more complex techniques in the Methods section.*
☐	☒	A description of all covariates tested
☐	☒	A description of any assumptions or corrections, such as tests of normality and adjustment for multiple comparisons
☐	☒	A full description of the statistical parameters including central tendency (e.g. means) or other basic estimates (e.g. regression coefficient) AND variation (e.g. standard deviation) or associated estimates of uncertainty (e.g. confidence intervals)
☐	☒	For null hypothesis testing, the test statistic (e.g. *F*, *t*, *r*) with confidence intervals, effect sizes, degrees of freedom and *P* value noted<br>*Give P values as exact values whenever suitable.*
☒	☐	For Bayesian analysis, information on the choice of priors and Markov chain Monte Carlo settings
☒	☐	For hierarchical and complex designs, identification of the appropriate level for tests and full reporting of outcomes
☒	☐	Estimates of effect sizes (e.g. Cohen's *d*, Pearson's *r*), indicating how they were calculated

Our web collection on statistics for biologists contains articles on many of the points above.

Software and code

Policy information about availability of computer code

Data collection	GraphPad Prism v8<br>SPSS v25<br>JMP Pro 14<br>Excel 64 bit<br>FCS Express v7<br>Image J
Data analysis	GraphPad Prism v8<br>SPSS v25<br>JMP Pro 14<br>Image J

For manuscripts utilizing custom algorithms or software that are central to the research but not yet described in published literature, software must be made available to editors and reviewers. We strongly encourage code deposition in a community repository (e.g. GitHub). See the Nature Portfolio guidelines for submitting code & software for further information.

Data

Policy information about availability of data

All manuscripts must include a data availability statement. This statement should provide the following information, where applicable:

- Accession codes, unique identifiers, or web links for publicly available datasets
- A description of any restrictions on data availability
- For clinical datasets or third party data, please ensure that the statement adheres to our policy

Source data for all figures in the report are available as a separate excel file. All protocols used in this study are available in the Methods and materials or Supplementary Methods sections. Clinical outcome, tumor mutation and gene expression data are publicly available from the Cancer Genome Atlas (TCGA).

Field-specific reporting

Please select the one below that is the best fit for your research. If you are not sure, read the appropriate sections before making your selection.

☒ Life sciences ☐ Behavioural & social sciences ☐ Ecological, evolutionary & environmental sciences

For a reference copy of the document with all sections, see nature.com/documents/nr-reporting-summary-flat.pdf

Life sciences study design

All studies must disclose on these points even when the disclosure is negative.

Sample size	Generally, in vitro studies were performed in 3-8 separate samples. In vivo studies were performed in 7-14 tumors per treatment arm. No a priori sample size determination was performed as generally these samples sizes (3 minimum for in vitro, 7 minimum for in vivo) were felt to be sufficient to evaluate treatment effect based our previous similar work.
Data exclusions	No data were excluded.
Replication	All attempts at replication were successful. Generally in vitro data (as well as the UMSCC47 in vivo study) was repeated twice at a minimum.
Randomization	In animal studies, tumors were randomly allocated once they reached ~150 mm3. For in vitro experiments, randomization has no expected benefit, as there is nothing to randomize against, as the cells all originate from the same source immediately prior to treatment.
Blinding	Blinding was not possible due to delivery of radiation in animal tumors. Two investigators were assigned to measure tumor volume to minimize bias. In regard to in vitro studies, blinding on a volume needed for each experiment would require more resources than are available in most laboratories and would provide minimal, if any, benefit for reasons similar to the lack of randomization.

Reporting for specific materials, systems and methods

We require information from authors about some types of materials, experimental systems and methods used in many studies. Here, indicate whether each material, system or method listed is relevant to your study. If you are not sure if a list item applies to your research, read the appropriate section before selecting a response.

Materials & experimental systems

n/a	Involved in the study	
☐	☒	Antibodies
☐	☒	Eukaryotic cell lines
☒	☐	Palaeontology and archaeology
☐	☒	Animals and other organisms
☐	☒	Human research participants
☒	☐	Clinical data
☒	☐	Dual use research of concern

Methods

n/a	Involved in the study	
☒	☐	ChIP-seq
☐	☒	Flow cytometry
☒	☐	MRI-based neuroimaging

Antibodies

Antibodies used

CCBP (D6C5) cst 1:2000 #7389
p300 (NM11) Santa Cruz 1:1000 sc-32244
BRCA1 (D-9) Santa Cruz 1:200 sc-6954
cleaved-caspase3 (Asp175)(5A1E) cst 1:500 #9664
Actin (C4) Millipore/Sigma 1:10,000 MAB1501
H3K9Ac (C5B11) cst 1:5000 #9649
H3K18Ac (D8Z5H) cst 1:5000 #13998

H3K27Ac (D5E4) cst 1:5000 #8173
total H3 (D1H2) cst 1:5000 #4499
Acetyl-lysine (RM101) Abcam 1:500 ab190479
53BP1 CST 1:200 #4937
p-Histone H2A.X (s139) 1:200 Cell Signaling Technology #2577
Acetyl-lysine affinity beads Cytoskeleton 50ul AAC04-beads
Protein A Sepharose beads GE healthcare 50ul, 100mg/ml 17-0780-01
ECL anti-rabbit IgG HRP GE healthcare 1:2000 NA934V
ECL anti-mouse IgG HRP GE healthcare 1:2000 NA931V
Cy3-conjugated donkey anti-mouse IgG Jackson ImmunoResearch 1:600 715-165-150 Cy3-conjugated donkey anti-rabbit IgG Jackson ImmunoResearch 1:600 711-165-152 FITC-conjugated donkey anti-mouse IgG Jackson ImmunoResearch 1:600 715-095-150 FITC-conjugated donkey anti-rabbit IgG Jackson ImmunoResearch 1:600 711-095-152

Validation

All antibodies used in this study are commonly used have been validated by the manufacturer, specifically for immunoblot and immunofluorescence where used for that purpose.

Eukaryotic cell lines

Policy information about cell lines

Cell line source(s)

HNSCC cell lines (UM-SCC-47, UM-SCC-22a, UM-SCC-25, UM-SCC-1, HN31, HN30, UM-SCC-17B, UPCI:SCC152, Cal-27, UD-SCC-2 and HN5) used in this study were generously supplied by Dr. Jeffrey Myers via The University of Texas MD Anderson Cancer Center Head and Neck cell line repository. HEK-293T, NCI-H520, NCI-H2228, NCI-H358, A549, Calu-6, FaDu and Detroit 562 were purchased form American Type Culture Collection (Manassas, VA).

Authentication

STR genotyping was performed prior to use for each cell line.

Mycoplasma contamination

Cell lines were tested for mycoplasma prior to use, any found to be positive were treated with antibiotics prior to use.

Commonly misidentified lines
(See ICLAC register)

None

Animals and other organisms

Policy information about studies involving animals; ARRIVE guidelines recommended for reporting animal research

Laboratory animals

Male athymic nude mice (6-8 weeks old, ENVIGO/HARLAN, USA)

Wild animals

Did not involve wild animals

Field-collected samples

Did not involve field-collected samples

Ethics oversight

In vivo studies were performed according to all relevant ethical regulations and following Institutional Care and Use Committee (IACUC) approval from both the University of Pittsburgh or The University of Texas MD Anderson Cancer Center depending on the individual experiment.

Note that full information on the approval of the study protocol must also be provided in the manuscript.

Human research participants

Policy information about studies involving human research participants

Population characteristics

This is the Head and Neck Cancer Genome atlas cohort (Hoadley et al. Cell, 2018 among others) which consists of de-identified head and neck squamous cell carcinoma tumors, and annotated clinical outcomes that are publicly accessible (at www.cbioportal.org or https://gdac.broadinstitute.org/ among other sites)

Recruitment

De-identified head and neck squamous cell carcinoma tumors were sent by participating institutions to the TCGA for analysis with the key criteria being available tissue.

Ethics oversight

This is a publicly available, de-identified database of information.

Note that full information on the approval of the study protocol must also be provided in the manuscript.

Flow Cytometry

Plots

Confirm that:

☒ The axis labels state the marker and fluorochrome used (e.g. CD4-FITC).

☒ The axis scales are clearly visible. Include numbers along axes only for bottom left plot of group (a 'group' is an analysis of identical markers).

☒ All plots are contour plots with outliers or pseudocolor plots.

☒ A numerical value for number of cells or percentage (with statistics) is provided.

Methodology

Sample preparation

TUNEL Assay
Following experimental treatments, all cells were collected including floating cells and TUNEL staining was performed using the APO-DIRECT Kit (BD Pharmingen) according to the manufacturer’s protocol. 500,000-1 million cells were fixed in 1% paraformaldehyde on ice for 30min. Cells were then washed in PBS and fixed in 70% ethanol overnight at -20C. Cells were washed twice with provided buffer then stained with 50µl of DNA labeling solution at 37°C for 45-60 min . Cells were then rinsed twice with provided buffer and resuspended in 300ul of rinse buffer. Cells were then analyzed by flow cytometry using the BD Accuri C6 Plus flow cytometer (BD Biosciences) with 488nm laser, 533/30 filter and FL1 detector. 10,000 events were measured per sample. Standard SSC and FSC gating were used to exclude debris. From the gated dot plot display, additional gating was applied at the edge of the unstained cell population (~4 log) and any events to the right of this population was gated as positive apoptosis (~5 log). 2µg/ml puromycin 24 and 48h, in addition to kit controls, were used as positive control samples to assist in proper delineation.

Cell cycle
24h after irradiation, all cells were collected including floating cells. Cells were then pelleted by centrifugation at 300 x g for 5min and washed with PBS twice, then fixed using 70% ethanol for at least 30min at 4oC or overnight at -20C. After fixation, cells were pelleted and washed once with PBS, then resuspended in propidium iodide 50ug/ml (Sigma Aldrich), 100ug/ml Rnase A (Sigma Aldrich) in PBS and incubated at room temperature for 30min. Samples were then analyzed by flow cytometry using the Accuri C6 Plus flow cytometer (BD Biosciences). Standard SSC and FSC gating were used to exclude debris. Standard gate was further gated by FL2-H and FL2-A, and additionally a third gating was applied, FL2-H by width, to remove doublets. A histogram was generated from these events and cell cycle distribution was quantified using FCS Express v7 using 1 cycle DNA fit analysis.

HR/NHEJ repair Assays
For GeneJet transfection, FaDu and HEK293T cells were plated into 10cm dishes. At 70% confluency cells were transfected (GeneJet, SignaGen) with 5µg pDRGFP (Addgene, Plasmid #26475) or pimEJ5GFP (Addgene, Plasmid 44026) and stably selected with 2µg/ml puromycin for two weeks. Stably selected cells were plated at 600,000 in 60mm dishes and incubated overnight. The next day cells were treated with 1.5µM A485/A486, 10µM ATMi (KU-55933) or 100nM ATRi (BAY-1895344) for 24h. The following day cells were transfected with 3µg pCBASceI (Addgene, Plasmid 26477) and 0.6µg mCherry (Addgene, Plasmid 41583) and incubated with drugs for a total of 72h. Flow cytometry was run using BD Accuri C6 Plus and standard SSC and FSC gating excluded debris. A dot plot display of FL1 (gfp) by FL2 (rfp) were gated for at the edge of negative control groups DRGFP or EJ5GFP. Any events to the right and upward from this gate were considered positive for repair. For electroporation, UMSCC22A, HN31, HN30, A549, H460, Calu6, H520, H2228 and H358 5µg pDRGFP (Addgene, Plasmid #26475) or pimEJ5GFP (Addgene, Plasmid 44026) were electroporated using Nucleofector 2b technology (Amaxa) (see Supplementary Table 7 for programs used for individual cell lines) and stably selected with 2µg/ml puromycin for two weeks. Stably selected cells were electroporated with 6µg pCBASceI (Addgene, Plasmid 26477) and 2µg mCherry (Addgene, Plasmid 41583) and incubated in 1.5µM A485/A486, 10µM ATMi (KU-55933) or 100nM ATRi (BAY-1895344) for a total of 72h. Flow cytometry was run using BD Accuri C6 Plus and standard SSC and FSC gating excluded debris. A dot plot display of FL1 (gfp) by FL2 (rfp) were gated at the edge of negative control groups DRGFP or EJ5GFP. Any events to the right and upward from this gate were considered positive for repair.

Instrument

BD Accuri C6 Plus flow cytometer (BD Biosciences)

Software

BD Accuri C6 Plus and FCS Express v7

Cell population abundance

Tunel assay: Standard SSC and FSC gating were used to exclude debris with 10,000 events evaluated. From the gated dot plot display, additional gating was applied at the edge of the unstained cell population (~4 log) and any events to the right of this population was gated as positive apoptosis (~5 log). 2µg/ml puromycin 24 and 48h, in addition to kit controls, were used as positive control samples to assist in proper delineation.

Cell cycle: Standard SSC and FSC gating were used to exclude debris with 10,000 events evaluated. Standard gate was further gated by FL2-H and FL2-A, and additionally a third gating was applied, FL2-H by width, to remove doublets. A histogram was generated from these events and cell cycle distribution was quantified using FCS Express v7 using 1 cycle DNA fit analysis.

HR/NHEJ Assays: Flow cytometry was run using BD Accuri C6 Plus and standard SSC and FSC gating excluded debris with 5,000 events analyzed. A dot plot display of FL1 (gfp) by FL2 (rfp) were gated at the edge of negative control groups DRGFP or EJ5GFP. Any events to the right and upward from this gate were considered positive for repair.

Gating strategy

Tunel assay: Standard SSC and FSC gating were used to exclude debris. From the gated dot plot display, additional gating was applied at the edge of the unstained cell population (~4 log) and any events to the right of this population was gated as positive apoptosis (~5 log). 2µg/ml puromycin 24 and 48h, in addition to kit controls, were used as positive control samples to assist in proper delineation.

Cell cycle: Standard SSC and FSC gating were used to exclude debris. Standard gate was further gated by FL2-H and FL2-A, and additionally a third gating was applied, FL2-H by width, to remove doublets. A histogram was generated from these events and cell cycle distribution was quantified using FCS Express v7 using 1 cycle DNA fit analysis.

HR/NHEJ Assays: A dot plot display of FL1 (gfp) by FL2 (rfp) were gated at the edge of negative control groups DRGFP or EJ5GFP. Any events to the right and upward from this gate were considered positive for repair.

☒ Tick this box to confirm that a figure exemplifying the gating strategy is provided in the Supplementary Information.
